# Identification of Sphingolipid Metabolites That Induce Obesity via Misregulation of Appetite, Caloric Intake and Fat Storage in *Drosophila*

**DOI:** 10.1371/journal.pgen.1003970

**Published:** 2013-12-05

**Authors:** Stanley M. Walls, Steve J. Attle, Gregory B. Brulte, Marlena L. Walls, Kim D. Finley, Dale A. Chatfield, Deron R. Herr, Greg L. Harris

**Affiliations:** 1 Department of Biology and Molecular Biology Institute, San Diego State University, San Diego, California, United States of America; 2 Bioscience Center, San Diego State University, San Diego, California, United States of America; 3 Department of Chemistry and Biochemistry, San Diego State University, San Diego, California, United States of America; 4 Department of Pharmacology, National University of Singapore, Singapore; National Cancer Institute, United States of America

## Abstract

Obesity is defined by excessive lipid accumulation. However, the active mechanistic roles that lipids play in its progression are not understood. Accumulation of ceramide, the metabolic hub of sphingolipid metabolism, has been associated with metabolic syndrome and obesity in humans and model systems. Here, we use *Drosophila* genetic manipulations to cause accumulation or depletion of ceramide and sphingosine-1-phosphate (S1P) intermediates. Sphingolipidomic profiles were characterized across mutants for various sphingolipid metabolic genes using liquid chromatography electrospray ionization tandem mass spectroscopy. Biochemical assays and microscopy were used to assess classic hallmarks of obesity including elevated fat stores, increased body weight, resistance to starvation induced death, increased adiposity, and fat cell hypertrophy. Multiple behavioral assays were used to assess appetite, caloric intake, meal size and meal frequency. Additionally, we utilized DNA microarrays to profile differential gene expression between these flies, which mapped to changes in lipid metabolic pathways. Our results show that accumulation of ceramides is sufficient to induce obesity phenotypes by two distinct mechanisms: 1) Dihydroceramide (C_14:0_) and ceramide diene (C_14:2_) accumulation lowered fat store mobilization by reducing adipokinetic hormone- producing cell functionality and 2) Modulating the S1P: ceramide (C_14:1_) ratio suppressed postprandial satiety via the hindgut-specific neuropeptide like receptor *dNepYr*, resulting in caloric intake-dependent obesity.

## Author Summary

Obesity is characterized by excessive weight gain that increases one's risk for pathologies such as Type II diabetes and heart disease. It is well-known that a high calorie diet rich in saturated fats contributes to excessive weight gain. However, the role that saturated fats play in this process goes far beyond simple storage in fat tissue. Saturated fats are essential building blocks for the bioactive lipid ceramide. Accumulation of ceramide has recently been associated with obesity. However, it is not known whether its accumulation plays an active role in the induction of obesity. Here, we utilized genetic manipulation in *Drosophila* to accumulate and deplete a variety of ceramide species and other related lipids. Our results showed that modulation of ceramide and related lipids is sufficient to induce obesity through two distinct mechanisms: a caloric intake-dependent mechanism works through suppression of neuropeptide Y satiety signaling, while a caloric intake-independent mechanism works through regulation of hormone producing cells that regulate fat storage. These data implicate ceramides in actively promoting obesity by increasing caloric intake and fat storage.

## Introduction

Obesity is a condition in which body weight, caused by excessive accumulation of stored body fat, is increased to the point where it becomes a risk factor for certain health conditions and mortality. Overweight and obese individuals are at an increased risk for hypertension, dyslipidemia, Type 2 diabetes, heart disease, stroke and certain forms of cancer. Unfortunately, obesity is a growing worldwide epidemic with over 1 billion of the global population either overweight or clinically obese. Our ability to understand the underlying mechanisms involved in the pathogenesis and progression of obesity are essential to developing new methods and approaches for combating this disease.

In the present study, we describe a central mechanistic role for sphingolipids (SL) in the progression of obesity. SLs are a versatile class of bioactive lipids, which play roles in a variety of signaling pathways that regulate diverse cellular functions such as programmed cell death, proliferation, migration, membrane stability, host-pathogen interactions and the stress response [1]–[4]. The basic structure of SLs consists of fatty acid chains linked by amide bonds to a long-chain “sphingoid” base. Biological functionality of each SL species can vary based on fatty acid chain length, degrees of saturation, and head group modification. Despite previous research detailing the cellular action of these lipids, their role at the organismal level and their homeostatic regulation *in vivo* is now just becoming understood with the emergence of suitable complex genetic models for analysis.

Ceramide, the metabolic hub of sphingolipid metabolism, has recently been associated with metabolic syndrome and obesity in humans as well as a variety of animal model systems [5]. For example, in obese insulin resistant humans, high levels of ceramide were detected in skeletal muscle tissue [5]. In obese leptin deficient *ob/ob* mice, ceramide levels were elevated in the serum [6]. Subsequent studies in these mice showed that pharmacological perturbation of *de novo* ceramide synthesis, using the serine palmitoyl-transferase inhibitor myriocin, induced weight loss and decreased fat storage [7]. This suggests that ceramide, and potentially other SL intermediates, are playing an active role in the pathogenesis of obesity. However, a gap in our knowledge still exists as to whether specific modulation of ceramide levels is sufficient to induce obese phenotypes.

Here, we use *Drosophila* as a model organism to determine whether *direct* perturbation of sphingolipid metabolism is sufficient to induce obese phenotypes. We used genetic manipulation to cause accumulation or depletion of ceramide intermediates, as well as to modulate the sphingosine-1-phosphate to ceramide ratio (also known as the S1P: ceramide rheostat). We demonstrate that genetic manipulations that cause direct ceramide accumulation induce obesity by two distinct mechanisms: 1) Dihydroceramide (C_14:0_) and ceramide diene (C_14:2_) accumulation lowered fat store mobilization by reducing adipokinetic hormone- producing cell functionality and 2) decreasing the S1P: ceramide (C_14:1_) ratio suppressed postprandial satiety via the hindgut-specific neuropeptide like receptor *dNepYr*.

## Results

### Blocking *de novo* synthesis of SLs lowers SL intermediate levels and promotes caloric intake-independent leanness

**Figure 1:**
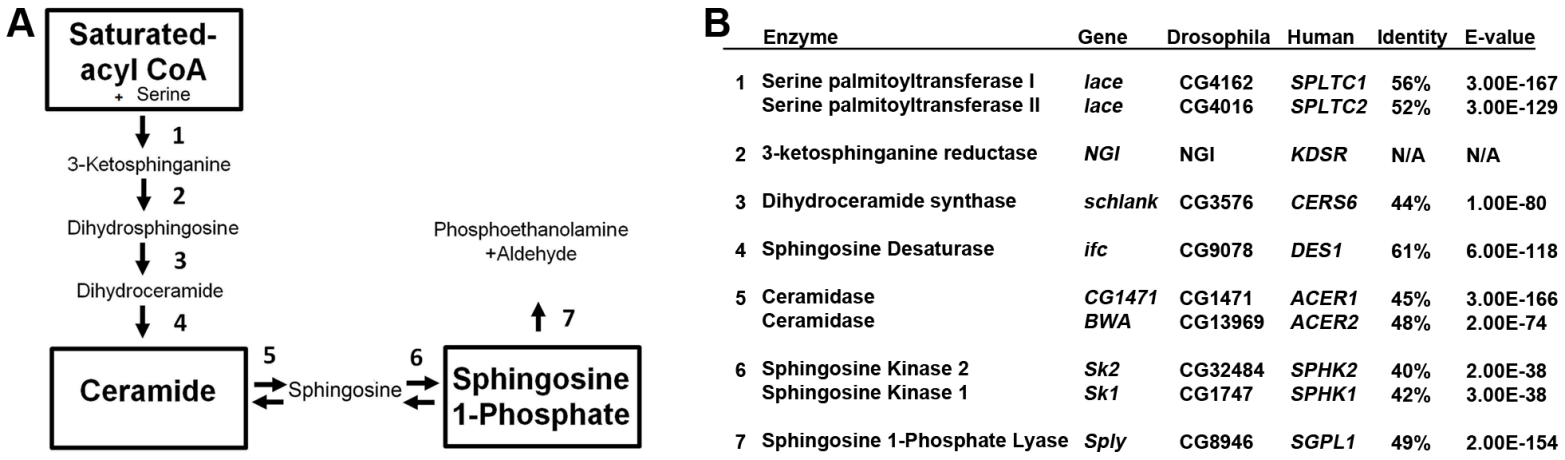
Sphingolipid metabolism in flies and humans. (A) *De novo* sphingolipid metabolism begins with the condensation of serine with palmitoyl-CoA catalyzed by serine palmitoyl transferase, which is encoded by *lace* (1). The resultant ketone is rapidly reduced by the actions of 3-ketosphinganine reductase (2) into dihydrosphingosine (DHS). The addition of a second fatty acid chain is carried out by ceramide synthase, encoded by the gene *schlank* (3), to produce dihydroceramide (DHC). DHC is desaturated by sphingosine delta 4 desaturase, encoded by *ifc* (4), producing ceramide. Ceramide can then be degraded by the actions of ceramidase (5) to form sphingosine. Either sphingosine or DHS can be phosphorylated by Sphingosine kinase 1 or 2 (6). Once phosphorylated, either can be irreversibly degraded by the actions of S1P lyase, encoded by *Sply*. (B) Protein encoding genes of the SL pathway are highly conserved between flies and humans. Identity and E-value determined using pBLAST analysis of *Drosophila* proteins from Flybase against Human database. (NGI = No gene identified).

The rate-limiting step of *de novo* SL synthesis is catalyzed by serine palmitoyl-transferase (SPT) (Figure 1A). In flies, SPT is encoded by the gene *lace*. Since homozygous null mutations of *lace* are lethal, we utilize transheterozygous *lace^k05305/2^* mutants to perturb *de novo* SL synthesis [8]. These mutants exhibit substantially reduced *lace* transcript levels (Figure S1A), with significant reductions in downstream SL intermediate levels, including total ceramide (−50%), sphingosine (−30%) and S1P levels (−48%) relative to wild type (wt) flies (Table 1).

**Table 1:** Sphingolipidomic profiles of SL mutants.

	Genotypes						
Sphingolipids	wildtype	lace	ifc	Sk2	Sply	lace/Sply	Ifc/Sk2
Sph C14:0	3.81±0.58	1.28±0.22*	4.99±0.42*	2.86±0.38*	7.93±0.96*	2.33±0.29*	2.85±0.45*
Sph C14:1	17.39±2.3	13.02±1.5*	14.83±1.9*	37.69±4.2*	95.89±6.9*	32.71±3.3*	22.29±2.8*
Sph C14:2	2.75±0.38	2.71±0.55	5.67±0.41*	3.16±0.64	32.30±1.6*	6.24±0.98*	5.17±1.1*
Sph C16:0	0.23±0.11	0.13±0.9	0.55±0.12*	0.10±0.07	0.33±0.09	0.27±0.15	0.36±0.12*
Sph C16:1	0.73±0.11	0.53±0.05	0.56±0.05	0.79±0.05	42.69±7.4*	0.32±0.12*	0.59±0.18
Sph Total	24.92±2.5	17.68±1.6*	26.60±2.1	44.59±4.9*	179.3±9.5*	41.87±3.5*	31.26±4.4*
S1P C14:0	0.54±0.14	n.d.*	1.93±0.98*	n.d.*	3.00±1.28*	n.d.*	0.98±0.01*
S1P C14:1	1.95±0.35	1.09±0.24	1.35±0.61	n.d.*	7.69±1.11*	5.23±0.95*	0.99±0.01*
S1P C16:0	0.50±0.09	0.30±0.05	0.95±0.15*	n.d.*	1.36±0.45*	1.38±0.45*	n.d.*
S1P C16:1	1.06±0.18	0.71±0.05	0.42±0.04*	n.d.*	2.48±0.29*	1.34±0.11	0.33±0.10*
S1P Total	4.05±0.75	2.11±0.44*	2.88±0.31	n.d.*	14.53±1.6*	7.94±1.01*	2.32±0.27*
C C14:0/C20:0	13.81±1.3	0.96±0.42*	37.0±3.6*	19.7±0.5*	12.4±0.9*	1.27±0.58*	43.7±4.9*
C C14:0/C22:0	41.0±9.0	0.44±0.03*	84.7±0.67*	36.7±6.0*	66.3±9.7	n.d.*	98.1±3.8*
C C14:0/C24:0	4.2±1.2	0.38±0.02*	3.5±0.03	4.7±1.9	9.9±4.0*	0.07±0.01*	10.0±0.38*
C C14:0 Total	59±6.0	1.78±0.47*	125.2±1.4*	61.1±2.9	88.5±5.8*	1.34±0.6*	151.8±6.5*
C C14:1/C20:0	538±40	331±13*	521±62	911±188*	746±96*	432±73	718±84*
C C14:1/C22:0	1415±148	772±33*	1285±72	1926±139*	2413±217*	1320±160	1765±146*
C C14:1/C24:0	262±34	82.7±3.5*	189±14*	1046±323*	542±36*	213±24	234±17
C C14:1 Total	2215±162	1186±49*	1995±108	3883±403*	3701±330*	1965±224	2717±242*
C C14:2/C20:0	12.6±0.7	1.31±0.01*	10.8±1.4	10.7±3.4	20.3±1.5*	2.67±0.82*	13.0±3.5
C C14:2/C22:0	30.1±2.7	13.7±0.1*	62.8±7.8*	11.6±2.1*	76.4±12.1*	9.32±1.3*	61.4±10.9*
C C14:2/C24:0	8.0±4.3	0.33±0.02*	2.67±0.88*	4.84±0.98*	9.7±0.5	1.47±0.15*	7.17±1.32
C C14:2 Total	50.7±3.7	15.4±1.1*	76.1±9.6*	27.1±1.6*	106.4±12*	13.5±2.2*	81.6±11.7*

**Sph = Sphingosine; S1P = Sphingosine 1-Phosphate; C = ceramide;** Dihydroceramide (C_14:0_), ceramide (C_14:1_) and ceramide diene (C_14:2_) subspecies are shown and represent the degree of saturation on the sphingoid backbone. C_20:0_, C_22:0_ and C_24:0_ denote the length and saturation of the second fatty acid chain connected to the sphingoid backbone in these ceramides.

* denotes p-value<0.05.

**Figure 2:**
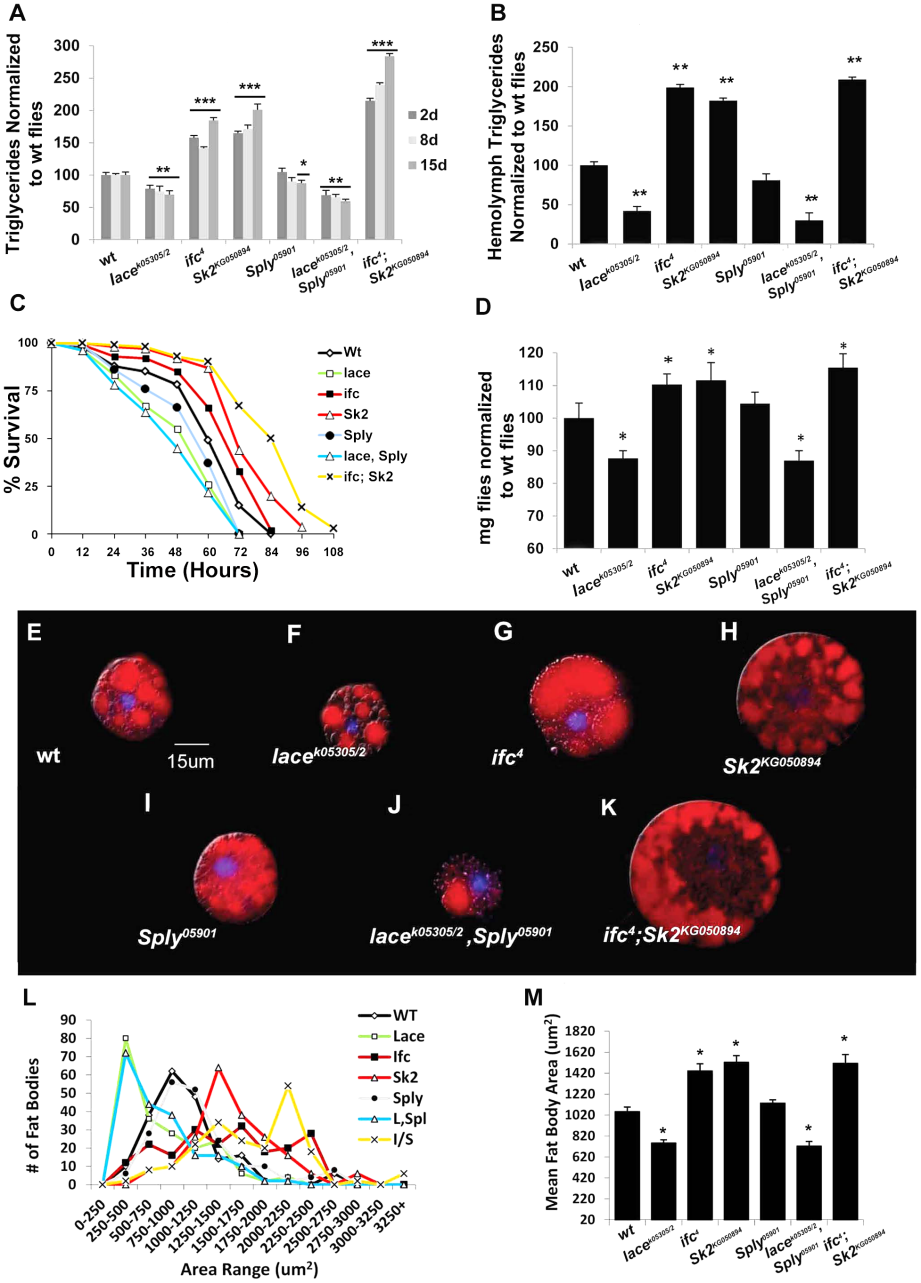
Classic hallmarks of obesity. Obesity in flies is characterized by classic hallmarks of obesity observed in higher organisms. (A) Whole fly triglyceride (TAG) and (B) hemolymph TAG levels were measured in µg of TAG per mg flies and normalized to wt flies. (C) Resistance to starvation-induced death was measured as the mean % of the population that survives over time of 3 independent experiments. (n = 100 flies) (D) Mean body weight (mg) was measured in six sets of n = 100 flies. (E–K) One-Day old larval origin fat body cells stained with lipid positive Nile Red (Red) and a nuclear stain DAPI (blue). (L) Distribution of fat body cell size (µm) from 50 randomly selected fat bodies from n = 4 flies used to calculate (M) mean fat body cell size. Error bars are represented by the S.E.M. p-values *<0.05, **<0.01, ***<0.001.

Ceramide-reducing *lace^k05305/2^* flies show significant reductions in whole fly triglyceride (TG) levels at 2 days (−21%), 8 days (−23%) and 15 days (−27%) of age relative to wt flies (Figure 2A). RNAi-mediated *lace* knockdowns showed comparable reductions in both *lace* transcript (Figure S1E) and mean whole fly TG levels (Figure S2B). Similarly, TG levels in hemolymph extracted from 2 day old flies were 58% lower than wt flies (Figure 2B, S2D). Survival under starvation on agar-only media was also perturbed in these flies, with a mean 50% survival time of 45 hours, compared to 60 hours in wt flies (Figure 2C). Mean body weight in 2 day old female flies was also reduced by 12% in *lace^k05305/2^* mutants (0.97 mg/fly vs.1.10 mg/fly in wt) (Figure 2D,S2C). Mutant *lace^k05305/2^* flies display fat body cell atrophy relative to wt flies (Figure 2F). Both mean cell (Figure 2L–M) and lipid droplet size (Figure S2E–G) were reduced. Abdominal sections of *lace^k05305/2^* mutants exhibited diminished adiposity characterized by less lipid positive staining then wt flies (Figure S3A, S3C). Collectively, we conclude that *lace^k05305/2^* mutants exhibit a lean phenotype relative to wt flies.

**Figure 3:**
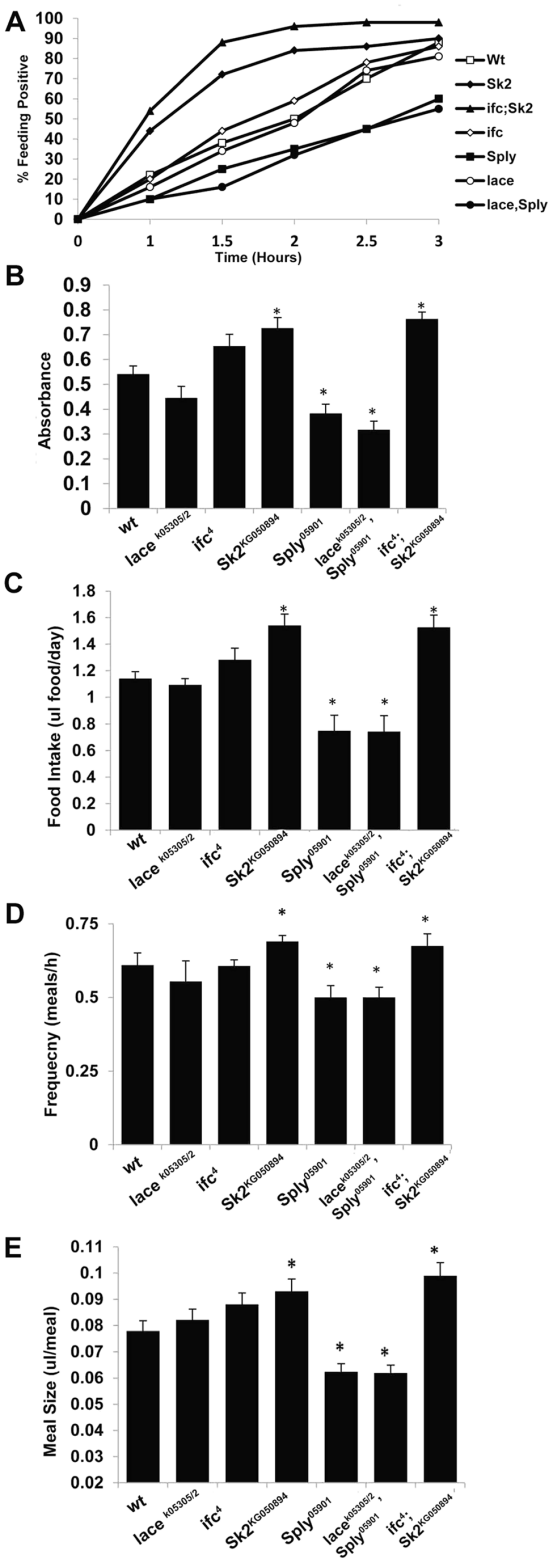
SL Metabolism and feeding behavior. Two different experimental approaches were used to determine appetite response, relative meal volume and mean daily food intake. (A) Flies starved for 3 hours where transferred to synthetic food dyed with 0.01% Bromophenol blue. Three sets of (n = 100) flies were scored for blue positive abdomens over time. The starvation-induced appetite response was plotted as % of fly population that fed overtime. (B) Flies from A that scored positive at 2.5 hours were collected, homogenized and evaluated for Bromophenol blue content by measuring absorbance at 545 nm. Absorbance is proportional to relative meal volume and shown as the mean of three sets of (n = 25) flies. The capillary feeding (CAFE) assay was performed to determine (C) mean daily intake of liquid food media per day and (D) the number of meals consumed per hour, which were used to calculate (E) the average meal volume. Error bars are represented by the S.E.M. *p-values<0.05, **<0.01.

Next, we utilized two independent feeding behavior assays to determine if the lean phenotype exhibited by *lace^k05305/2^* mutants was dependent on changes in caloric intake. First, flies were subjected to 3 hours of starvation on agar-only media, and then transferred to Bromophenol blue stained food. The relative starvation-induced appetite response of flies was quantified as the percentage of flies which scored positive for feeding (blue abdomens) over time [9]. Second, flies were monitored in a capillary feeding (CAFE) chamber that allowed us to determine mean daily food intake and meal frequency [10]. No significant changes in starvation-induced appetite (Figure 3A) or postprandial meal volume (Figure 3B) were observed between *lace^k05305/2^* mutants and wt flies. Furthermore, there were no differences in mean daily caloric intake (Figure 3C), mean meal frequency (Figure 3D) or mean meal volume (Figure 3E). Taken together, we conclude that *lace^k05305/2^* mutants exhibit a caloric intake-independent lean phenotype.

### Sphingosine Δ-4 desaturase mutants exhibit perturbations in ceramide subspecies and caloric intake-independent obesity

Downstream of *lace* and *schlank* (Figure 1A–B), *ifc* encodes the enzyme sphingosine Δ-4 desaturase, which catalyzes the conversion of dihydroceramide into ceramide (Figure 1A). Mutant *ifc* ^4^ flies exhibit a 55% reduction in *ifc* transcript (Figure S1B), and significant changes in the *subspecies* of each SL. Specifically, *ifc^4^* mutant flies accumulate the C_14:0_ “dihydro” (DH) subspecies, with C_14:0_ dihydroceramide (DHC), dihydrosphingosine (DHS) and dihydrosphingosine 1-phosphate (DHS1P) intermediate levels increased +306%, +83%, and +257% respectively. Conversely, levels of monounsaturated C_14:1_ ceramides (−10%), sphingosine (−15%) and S1P (−31%) SL intermediates were reduced. Notably, these lines also accumulate the polyunsaturated C_14:2_ ceramide “diene” (+190%) and sphingosine “diene” (+206%) [11].

Mutant *ifc^4^* flies exhibit increased whole fly TG at 2 days (+58%), 8 days (+50%) and 15 days (+85%) (Figure 2A). Both transheterozygous *ifc^4^* mutants over deficiency and global *ifc* RNAi mediated knockdowns (Figure S1F) exhibit similar increases mean TG levels (Figure S2A–B). Hemolymph TG levels were also increased (+95%) (Figure 2B, S2D). Survival under starvation was enhanced, with a mean 50% survival time of 68 hours, compared to 60 hours in wt flies (Figure 2C). Mean body weight was also increased by 9% (1.20 mg/fly vs.1.10 mg/fly in wt) (Figure 2D, S2C). These flies also exhibit fat body cell hypertrophy (Figure 2G, 2L–2M), increased fat body lipid droplet size (Figure S2E–G), and increased abdominal adiposity (Figure S3E). Collectively, we conclude that *ifc^4^* mutants exhibit an obese phenotype relative to wt flies.

In *ifc^4^* mutants, the starvation-induced appetite response was slightly increased relative to wt flies (Figure 3A). However, no significant change in relative post prandial meal volume was observed (Figure 3B). Furthermore, *ifc^4^* mutants do not exhibit significant changes in mean daily caloric intake, mean meal frequency or mean meal volume (Figure 3C–3E) in the CAFE relative to wt flies. Taken together, we conclude that *ifc^4^* mutants exhibit a largely caloric intake-independent obese phenotype.

### Sphingosine kinase 2 mutants accumulate ceramide and exhibit caloric intake-dependent obesity

The *Sk2* gene encodes for the enzyme sphingosine kinase 2 (Figure 1A), which phosphorylates sphingosine into S1P. *Sk2^KG050894^* mutants exhibit an approximate 80% reduction in *Sk2* transcript with substantial increases in total sphingosine (+79%) and total ceramide (+55%) levels. Conversely, S1P levels are undetectable using our method in these flies (Table 1).

Mutant *Sk2^KG050894^* whole fly TG levels increased at 2 days (+65%), 8 days (+72%) and 15 days (+101%) (Figure 2A). Both transheterozygous *Sk2^KG050894^* mutants over their deficiency and *Sk2* RNAi mediated knockdowns (Figure S1F) exhibit a similar increase in TG levels (Figure S2A–B). Increased TG levels are also observed in hemolymph (+75%) (Figure 2B, Figure S2D). Survival under starvation was enhanced, with a mean 50% survival time of 71 hours (Figure 2C). Mean body weight was increased by 10% (1.21 mg/fly vs.1.10 mg/fly in wt) (Figure 2D, Figure S2C). These results correlate with observed fat body cell hypertrophy (Figure 2H, 2L–2M), increased mean lipid droplet size (Figure S2E–F), and increased abdominal adiposity (Figure S1A, S1F). Collectively, we conclude that *Sk2^KG050894^* mutants exhibit an obese phenotype relative to wt flies.

Obese *Sk2^KG050894^* mutants exhibit a substantial increase in starvation-induced appetite response, where over 50% of these flies consumed food within the first hour post-starvation relative to just 15% of control flies (Figure 3A). Furthermore, relative postprandial meal volume was increased (+34%) in *Sk2^KG050894^* mutants relative to wt flies (Figure 2B). Additionally, *Sk2^KG050894^* mutants consumed an average of 34.7% more food per day than controls (1.55 ul/day vs.1.15 ul/day) (Figure 3C), exhibiting increases in both meal frequency (Figure 3D) and size (Figure 3E). Based on these results, we conclude that ceramide accumulating *Sk2^KG050894^* flies exhibit caloric intake-dependent obesity.

### Sphingosine 1-phosphate: An opposing role in energy homeostasis

The committal step of SL degradation is catalyzed by S1P lyase, encoded by *Sply*, which irreversibly degrades S1P (Figure 1A). *Sply^05901^* mutant flies show substantial loss of *Sply* transcript (Figure S1D), and accumulate all SL intermediates, especially S1P (+260%) (Table 1).

Young *Sply^05901^* mutant flies exhibit a lipid metabolic phenotype similar to that observed in wild-type flies, with no significant changes in 2- and 8- day whole fly TG levels, hemolymph TG levels (Figure 2A–2B, Figure S2D), or body weight (Figure 2D, Figure S2C). Transheterozygous *Sply^05901^* mutants over their deficiency and global *Sply* RNAi knockdowns (Figure S1H) exhibit comparable whole fly TG levels (Figure S1A–B). This correlates with no observable change in mean fat body cell size (Figure 2I, 2L–M), lipid droplet size (Figure S2E–F) or abdominal adiposity (Figure S3B).

However, 2 day old *Sply^05901^* mutants display a decrease in their starvation-induced appetite response (Figure 3A) with a concomitant 30% reduction in relative starvation-induced postprandial meal volume (Figure 3B). Furthermore, these flies consumed significantly lower quantities of food (−35%) per day in the CAFE (Figure 3C), which was the result of a decrease in both meal frequency (Figure 3D) and size (Figure 3E). Interestingly, these results correlate with a reduction in starvation resistance (Figure 2C) This is possibly the result of fewer flies, on average, entering the starvation chambers in the fed state (and with a lower postprandial meal volume). A 21% reduction in TG levels is observed by 15 days (Figure 2A). This suggests that reduced caloric intake observed at 2 days leads to a reduction in TG stores, sometime between 8 and 15 days. In *Sply^05901^* flies, where ceramide levels are elevated in the context of high S1P levels, it appears that the classic hallmarks of obesity associated with high ceramide levels, are mitigated through reduced caloric intake.

### Double mutants combine components of caloric intake- independent and dependent leanness and obesity

Based on our results, the SL metabolites appear to regulate global energy metabolism by both caloric intake- independent and -dependent mechanisms. To understand this, we utilized two double mutant models that we hypothesized would combine components of each phenotype.

The first double mutant, *lace^k05305/2^; Sply^05901^* (Figure S1A, S1D) combined two mutations associated with reduced fat storage and reduced caloric intake respectively. If these mechanisms are distinct, then their effects should be additive and the double mutants should display an exacerbated lean phenotype. Mutant *lace^k05305/2^; Sply^05901^* flies exhibit a substantial reduction in dihydroceramide (−97%) and ceramide diene levels (−73%), as observed in *lace^k05305/2^* flies (Table 1). These double mutants also exhibit a large increase in S1P levels (+40%) relative to wt flies, similar to *Sply^05901^* mutants (Table 1). In this respect, we have shifted the S1P: ceramide ratio by both increasing S1P levels and reducing ceramides.

As predicted, *lace^k05305/2^; Sply^05901^* double mutants exhibit an exacerbated lean phenotype. Whole fly TG levels decreased at 2 days (−31%), 8 days (−40%) and 15 days (−47%) (Figure 2A). This correlated to a substantial reduction in starvation resistance (42 hrs. vs. 60 hrs.), body weight (−11%) (Figure 2C–2D, Figure S2C), and abdominal adiposity (Figure S1A, S1D). Fat body cell atrophy (Figure 2J, 2L–2M) was also observed, with a marked reduction in mean lipid droplet size (Figure S2E–2G). Furthermore, *lace^k05305/2^; Sply^05901^* double mutants exhibit a reduction in their starvation-induced appetite response with reduced post prandial meal volume (Figure 3A–3B). These flies also displayed reduced mean daily food intake, meal frequency and meal volume in the CAFE (Figure 3C–3E).

The second double mutant, *ifc^4^; Sk2^KG050894^*, (Figure S2B–C) combines two mutations associated with caloric intake-independent and dependent obesity. Again, if the mechanisms are distinct, the effects should be additive and in this case, these flies should display an exacerbated obese phenotype. Mutant *ifc^4^; Sk2^KG050894^* flies exhibit increases in dihydroceramides (+223%) and ceramide dienes (+62%), as observed in *ifc^4^* flies (Table 1). Simultaneously, total S1P levels are decreased (−43%), including undetectable levels of C_14:1/16:1_ S1Ps as observed in *Sk2^k6050894^* flies (Table 1). In this respect, we have shifted the ceramide: S1P rheostat towards ceramide, by both increasing ceramide levels while simultaneously decreasing S1P levels.

As predicted, these modulations of SL intermediate levels resulted in *ifc^4^; Sk2^KG050894^* mutants exhibiting an exacerbated obesity phenotype. Whole fly TG levels were elevated at 2 days (+115%), 8 days (+140%) and 15 days (+184%) (Figure 2A). This correlated to a substantial increase in starvation resistance (84 hrs. vs. 60 hrs.), body weight (+14%) (Figure 2C–2D, S2C), and abdominal adiposity (Figure S1G). Fat body cells were hypertrophied (Figure 2K–M), with elevated mean lipid droplet size (Figure S2E–2G). Furthermore, *ifc^4^; Sk2^KG050894^* double mutants exhibit increases in their starvation-induced appetite response and elevated starvation-induced post prandial mean meal volume (Figure 3A–3B). These flies display elevated mean daily food intake, meal frequency and meal volume (Figure 3C–3E).

### Caloric intake-independent leanness and obesity are associated with changes in pro and anti-apoptotic gene expression

**Figure 4:**
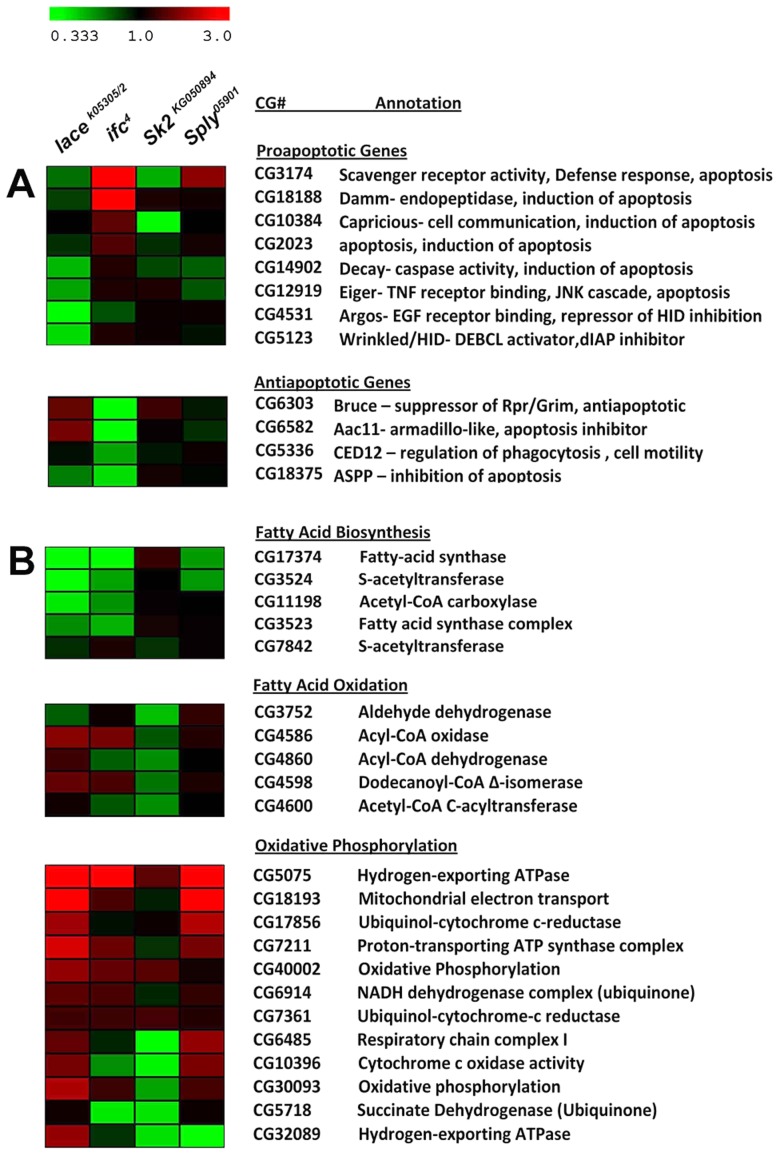
Differential expression of Lipid Metabolic and Apoptotic genes in SL mutants. DNA microarray analysis in conjunction with DAVID bioinformatics analysis was used to identify distinct subsets of genes mapped to elucidated pathways. Downregulation (Fold change <1.5) is shown in green while upregulation (Fold change >1.5) is shown in red, up to a maximum of 3-fold or greater difference. All changes >3 fold are represented by the brightest color. No change is displayed as black. (A)These data show that diene-accumulating, Akh cell-ablating *ifc^4^* mutants exhibit upregulation of proapoptotic genes and downregulation of anti-apoptotic genes, while diene-depleting, Akh cell-expanding *lace^k05305/2^* mutants' exhibit downregulation of proapoptotic genes and upregulation of anti-apoptotic genes. (B) These data show that appetite suppressed S1P accumulating *Sply^05901^* mutants' downregulate lipogenic pathways (FA biosynthesis) and upregulate lipid utilizing pathways (Fatty acid Oxidation and Oxidative Phosphorylation). Conversely, high appetite, S1P depleting *Sk2^KG050894^* mutants upregulate lipogenic pathways (specifically FA synthase) and downregulate lipid utilizing pathways.

In order to correlate the sphingolipidomic profile of our mutants to changes in metabolic phenotypes, we performed a DNA microarray analysis of these mutants across 14,000+ *Drosophila* transcripts of the *Drosophila* genome. Heatmap and DAVID analysis revealed differential expression of apoptosis-related genes between *lace^k05305/2^* and *ifc^4^* mutants. Analysis showed that specific subsets of proapoptotic genes were downregulated while anti-apoptotic genes were upregulated in *lace ^k05305/2^* flies (Figure 4A). Conversely, in the same gene subsets, *ifc^4^* mutants showed increased expression of pro-apoptotic genes and decreased expression of anti-apoptotic genes (Figure 4A).

**Figure 5:**
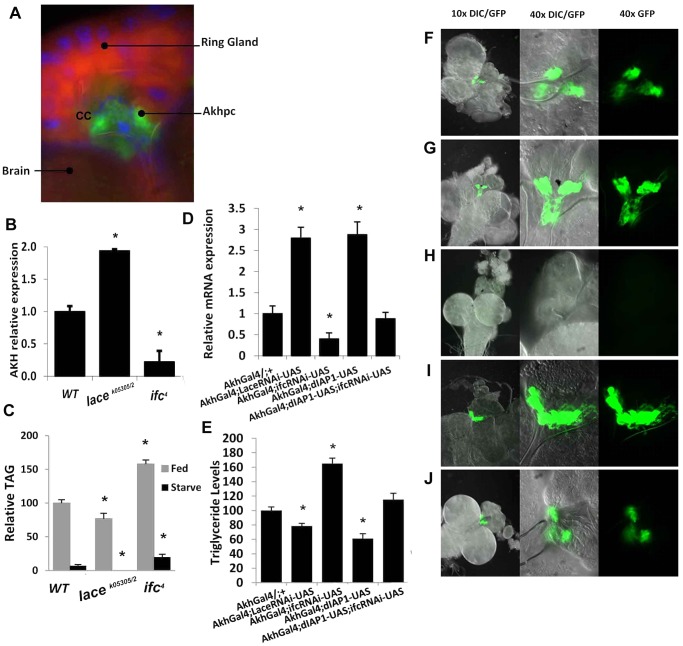
Caloric intake-independent obesity via regulation of Akhp cell viability and function. (A) Adipokinetic hormone-producing cells (Akhpc), (gfp) make up most of the corpus cardiac (cc) of the ring gland, located near the brain (dapi/blue = nuclear, nile red = counterstain). (B) *Akh* mRNA, which is only expressed in Akhp, is upregulated in *lace^k05305/2^* and downregulated in *ifc^4^* mutants. (C) Fat mobilization in starved *ifc^4^* flies is incomplete before starvation-induced expiration, while no remaining TAG stores are detectable in *lace^k05305/2^* flies. (D) Akhpc-specific RNAi mediated knockdown of *lace* and *ifc* phenocopy whole knockout *Akh* expression levels in 2 day old flies, with similar changes in (E) TAG level (control (AkhGal4/+;+) while Akhpc-specific overexpression of dIAP1 (inhibitor of apoptosis) mitigates the effects of *ifc* knockdown (Akh;UAS-*dIAP1*;UAS-*ifc*-rnai). These effects can be visualized in green (GFP) third instar larval Akhp cells, in Akhpc-specific (F) control (AkhGal4;UAS-GFP) (G) RNAi-mediated *lace* knockdowns (AkhGal4;UAS-*lace*-rnai), (H) RNAi-mediated *ifc* knockdowns (AkhGal4;UAS-*ifc*-rnai) (I) *dIAP1* overexpression (AkhGal4;UAS-*dIAP1*) and (J) dIAP rescue of ifc knockdowns (AkhGal4;UAS-*dIAP1*;UAS-*ifc*-rnai) Error bars are represented by the S.E.M.. *p-values<0.05, **<0.01.

Recently, apoptosis-induced cell death of Adipokinetic hormone-producing cells (Akhpc) was shown to increase TG storage [12]. Akhpc make up the majority of the cells in the corpus cardiac (CC) located in the brain (Figure 5A). The Akh peptide activates mobilization of TG stores from the fat body [12]. Interestingly, *lace^k05305/2^* and *ifc^4^* mutants display differential expression of the gene *Adipokinetic hormone* (*Akh*). Hence, we hypothesized that regulation of apoptosis in Akhpcs might be involved in caloric intake-independent phenotypes.

Akhpc-ablated flies display reduced *Akh* mRNA expression, elevated TG levels, live longer under starvation and exhibit five times higher TG levels after starvation-induced death relative to control flies [12]. Similarly, *ifc^4^* flies exhibit reduced *Akh* mRNA expression (Figure 5B), elevated TG levels (Figure 2A), live longer under starvation (Figure 2C) and exhibit elevated TG levels after starvation-induced death (Figure 5C). The opposite effect is observed in *lace^k05305/2^* flies which showed a nearly 2 fold increase in *Akh* mRNA expression (Figure 5B), reduced TG levels (Figure 2A), a shortened life span under starvation (Figure 2C) and undetectable post-starvation TG levels (Figure 5C).

### Caloric intake-independent leanness and obesity are regulated by adipokinetic hormone-producing cells

We utilized a GAL-4/UAS system to achieve Akhpc-specific RNAi mediated knockdown of *lace* and *ifc* mRNA. Akhpc-specific *ifc* knockdown (Akh-g4/ifc RNAi) was sufficient to induce a significant reduction in *Akh* transcript expression; with a concomitant increase in adult fly TG levels (Figure 5D–5E). The magnitude of *Akh* mRNA loss and TG elevation was similar to those observed in *ifc^4^* mutants (Figure 5B, Figure 2B) and global *ifc* RNAi knockdowns (Figure S1F, S2B, S5F). This suggests that *ifc* regulates Akhpc function in an Akhpc autonomous manner.

Akh-g4/ifc RNAi 3^rd^ instar larvae also exhibited decreased expression of GFP in Akhp cells relative to controls, as observed in both the cell body and neuronal projections (Figure 5F, 5H). In 70% of 3^rd^ instar larvae, undetectable levels of GFP expression were observed, while the remaining 30% showed only very low levels of GFP expression (Figure S6A, S6C, S6F). Notably, GFP expression was generally relegated to rounded cell bodies, with an absence of GFP-expressing neuronal projections. Semi-quantification of GFP expression in those larvae showed a reduction in both mean GFP positive area and optical density relative to control flies (Figure S6G). These data correlated with a substantial reduction in *Akh* mRNA levels in Akh-g4/ifc RNAi 3^rd^ instar larvae (Figure S6H).

Next, we examined Akhpc-specific knockdown of *Akh* itself (Akh-g4/Akh RNAi), to determine if the loss of GFP expression might be due to inhibition of Akh-g4 expression in these constructs. All Akh-g4/Akh RNAi 3^rd^ instar larvae examined expressed detectable levels of GFP (Figure S6A–B, S6F), in spite of substantially reduced Akh mRNA (Figure S6H). However, while mean GFP positive area was comparable to controls, a significant reduction in mean optical density was observed, suggesting that some GFP production is likely perturbed in these lines (Figure S6G).

These data suggest that the absence/reduction of GFP expression in Akhpc of Akh-g4/ifc RNAi 3^rd^ instar larvae is not due to complete inhibition of GFP expression, but rather is due to the absence/perturbation of Akhpc. Previous reports have shown that ablation of Akhp cells can be carried out via overexpression of the genes *Grim*, *Hid* and *Reaper*, which utilizes activation of the caspase-dependent intrinsic apoptotic pathway to induce Akhp cell death [12]. These proteins are known inhibitors of the *Drosophila* Inhibitor of Apoptosis Protein1 (dIAP1), which inhibits the activity of proapoptotic caspases Dronc and Drice. If *ifc* knockdown induced Akhpc ablation, we hypothesized that overexpression of *dIAP1* should rescue Akhp cell death as well as normalize reduced *Akh* mRNA and elevated TG levels.

Akhp cell specific exogenous expression of *dIAP1* (Akh-g4/+; UAS dIAP1/+) increased *Akh* transcript expression and decrease TG levels relative to control flies (Figure 5D–5E). In 3^rd^ instar larvae, GFP expression was elevated as observed in both the cell body and axonal projections. (Figure 5F, 5I). All Akh-g4/+;UAS dIAP1/+ 3^rd^ instar larvae exhibited robust GFP expression, with marked elevations in both mean GFP positive area and optical density (Figure S6A,S6D,S6F–G). These data correlated with a ∼2 fold increase in 3^rd^ instar larval *Akh* mRNA expression (Figure S6H). These findings provide strong evidence that Akhp cells are sensitive not only to induction, but also inhibition of the caspase-dependent apoptotic pathway during the larval stages of development.

Furthermore, Akhpc specific overexpression of *dIAP*, in an Akhpc-specific *ifc* knockdown background, partially rescued Akhp cell viability and function. In 3^rd^ instar larvae, although GFP expression was slightly less than in wt larvae, it was observable in 100% of samples (Figure 5F, 5J). This correlated well with *Akh* mRNA and TG levels in these flies, where no significant change relative to wt flies was observed. (Figure 5D–5E).

Akhp cell specific *lace* knockdowns (Akh-g4/+; *lace* RNAi/+) (Figure 5D–5G), exhibited reductions in 2 day fly *Akh* mRNA (Figure 5D) and whole fly TG levels (Figure 5E). In 3^rd^ instar larvae, Akh GFP-expression was enhanced, with an observable increase in cell density and neuronal projections (Figure 5G,S6A,SF6E). This observation could be attributed to an increase in both mean GFP+ Akhp cell area and optical density (Figure S6A,S6E,S6F–G). These data correlated well with a marked increase in *Akh* mRNA levels, as was similarly seen in Akh-g4/+; UAS dIAP1/+ 3^rd^ instar larvae. (Figure S6H).

### The role of S1P in Akhp cell regulation

Some data suggest that *Sply* may play a role in Akhp cell viability and function. First, expression levels of *Akh* mRNA in *Sply* ***^05901^*** are slightly reduced compared to wild-type flies (Figure S5). Secondly, *Sply* ***^05901^*** flies exhibit slightly elevated TG levels at 2 days (+9%). However, these flies show no significant difference in post-starvation TG levels, suggesting that fat mobilization is largely unperturbed (Figure S5).

Interestingly, Akhpc-specific *Sply* knockdowns (Akh-g4/*Sply* RNAi) exhibit an increase in TG levels (+40%) (Figure S2B) that correlates with a 50% reduction in *Akh* mRNA expression (Figure S5D). These effects were not observed in global *Sply* knockdowns (Figure S2B, S5D), suggesting that systemic factors (possibly systemic S1P pools) prevent loss of Akhpc viability and function.

### Caloric intake-dependent mechanisms are associated with changes in subsets of lipid metabolic genes

Next, we compared changes in global gene expression between caloric intake-dependent obese *Sk2^KG050894^* flies and lean *Sply^05901^* flies. Heatmap and DAVID analysis showed opposing expression in gene subsets regulating major lipid metabolic pathways, including fatty acid biosynthesis and oxidation, as well as oxidative phosphorylation (Figure 4B). *Sply^05901^* mutants exhibit downregulation of key fatty acid biosynthesis genes while showing upregulation of fatty acid oxidation and oxidative phosphorylation genes, suggesting a shift in basal gene expression towards a low energy, fat-burning, “unfed” state. Conversely, *Sk2^KG050894^* flies exhibit upregulation of the FA biosynthetic gene fatty-acid synthase, with downregulation of fatty acid oxidation genes and oxidative phosphorylation genes (Figure 4B). This suggests a shift in basal gene expression towards a high energy, fat-storing, “fed” state.

Additionally, differential expression was observed for *CG5811*, which encodes for the neuropeptide Y-like receptor (dNepYr) (Figure S4A) [13]. A set of pancreatic TG lipases appear to be *cis*-natural antisense transcripts (*cis*-NATs) of *dNepYr* in full overlapping orientation (Figure S4A) [13]. In adult flies, *dNepYr* transcript has been observed to be expressed almost exclusively in the hindgut (Figure S4B), with lower levels of expression observed in the brain [13], [14]. Mammalian neuropeptide Y receptors (NPYR) with enriched expression in the digestive system are generally associated with induction of post-prandial satiety and include NPYR 2 and 4 [15]. The *dNepYr* gene is expressed throughout development starting in early embryo(4–6 hr), with peak expression seen in late embryos (16 hr–24 hr) and throughout adulthood (Figure S4C) [16].

**Figure 6:**
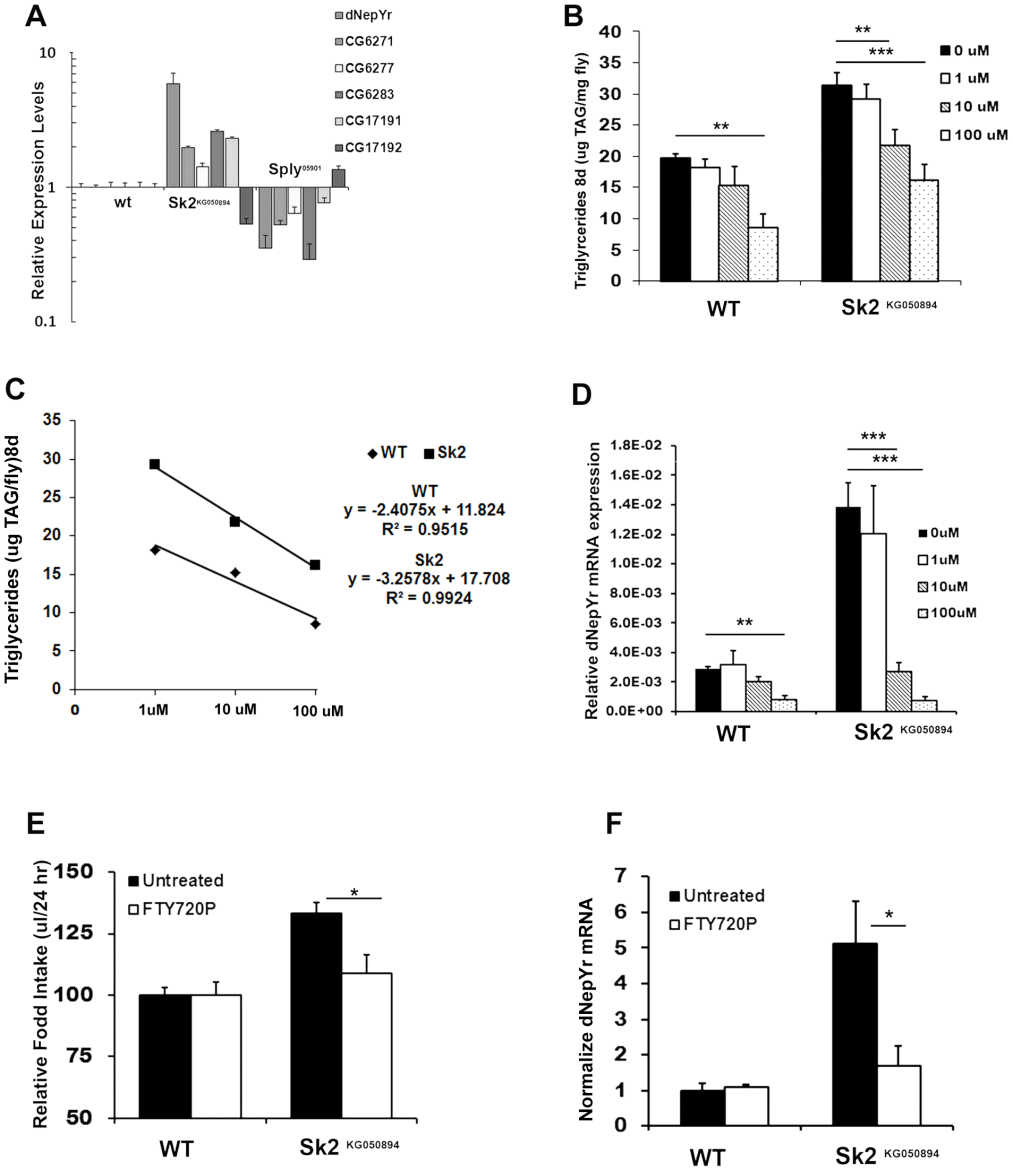
The ceramide:S1P rheostat's role in regulating *dNepYr* expression and caloric intake. (A) *dNepYr* expression is upregulated in *Sk2^KG6050894^* and downregulated in *Sply^05901^* mutants, with concurrent changes in overlapping cis-NAT's of pancreatic TAG lipase genes. Two day old *Sk2^KG6050894^* flies and wt flies were administered 0 uM, 1 uM, 10 uM and 100 uM dRYamide (1∶1 dRYamide 1∶2) in solid food for 6 days, after which (B) TG levels, (C) rate of dose dependent TG decline, and (D) *dNepYr* mRNA levels were measured. Three to five day old *Sk2^KG6050894^* flies were also administered S1P analogue FTY720P in the CAFE, after which (E) caloric intake (F) and *dNepYr* mRNA levels were measured. Error bars are represented by the S.E.M. p-values<*0.05, **<0.01, ***p<0.001.

Interestingly, *dNepYr* is upregulated in *Sk2^KG050894^* and downregulated in *Sply^05901^* mutants (Figure 6A). Its expression also appears to be coordinated with *cis*-NAT pancreatic TG lipase transcripts, and likely constitutes a novel system of coordinated dietary TG degradation and satiety signaling (Figure 6A, Figure S4A Therefore, we hypothesized that dNepYr signaling is associated with induction of satiety. To test this, we utilized RNAi-mediated *dNepYr* knockdown flies (Figure S4D). These flies exhibit increased mean daily caloric intake (Figure S4F) with a concomitant increase in TG levels (Figure S4F). These data suggest that dNepYr signaling is involved in post-prandial satiety.

The recently characterized *Drosophila* gene *CG40733* was found to encode two dRYamides that are expressed exclusively in the brain and the gut in *Drosophila* [17]. Both dRYamide1 and dRYamide2 were found to be strong ligands of dNepYr that negatively regulate feeding behavior [17]. Therefore, we examined the effects of dRYamide loss by characterizing *CG40733* RNAi knockdowns. Global *CG40733* RNAi knockdowns (Figure S4G) exhibit increases in both caloric intake (Figure S4H) and TG levels (Figure S4I).

### Caloric intake-dependent mechanisms are regulated by *dNepYr*, a negative regulator of feeding behavior

Based on these findings, we designed experiments to pharmacologically rescue elevated TG levels in *Sk2^KG050894^* mutants by administering synthetic dRYamide1 and dRYamide2 peptides (1∶1). *Sk2^KG050894^* mutants were more responsive to a lower concentration of dRYamides then wt flies. At 10 uM dRYamides, *Sk2* mutants exhibited a reduction in TG levels relative to 0 uM fed *Sk2^KG050894^* mutants, and were not statistically different to control flies fed either 0 uM or 10 uM dRYamide containing food (Figure 6B). The dose dependent rate of TG decline in *Sk2^KG050894^* flies was steeper (m = −3.26, R^2^ = 0.9924) than wt flies (m = −2.41, R^2^ 0.95) (Figure 6C). Furthermore, 10 uM and 100 uM dRYamide-fed *Sk2^KG050894^* flies also exhibit a concomitant reduction in *dNepYr* expression to near control levels (Figure 6D).

Reductions in TG levels and *dNepYr* mRNA levels (Figure 6D) were also observed in wt flies administrated 100 uM dRYamide (Figure 6B). At 100 uM dRYamide, wild type and *Sk2^KG050894^ dNepYr* mRNA levels were equally suppressed. However, 100 uM dRYamide-fed *Sk2* mutants' exhibit significantly higher levels of TG relative to 100 uM dRYamide-fed wt flies, suggesting that increased caloric intake through perturbation of *dNepYr* expression is not the only contributing factor to the obesity phenotype.

**Figure 7:**
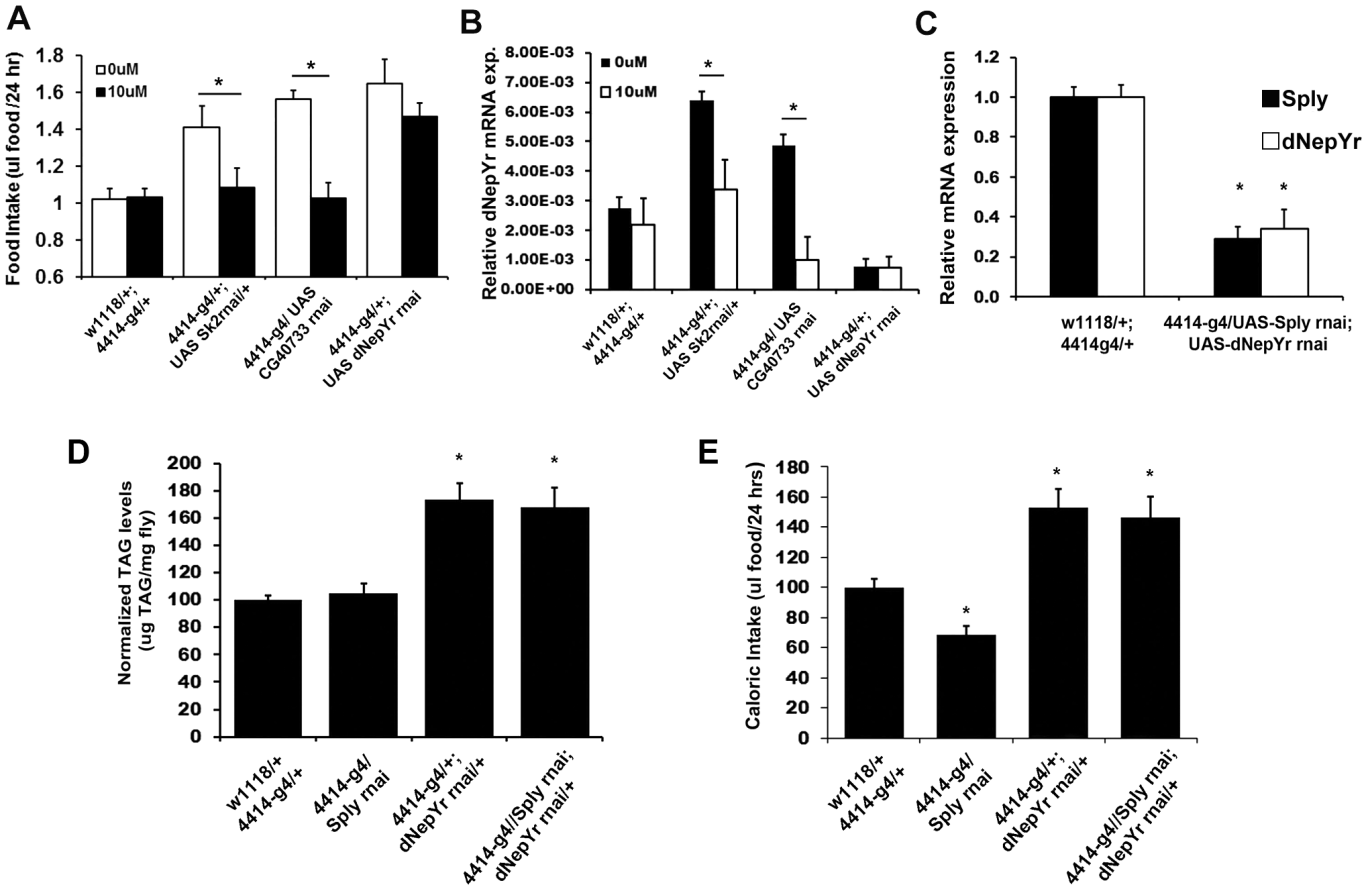
Genetic and pharmacological modulation of *dNepYr* expression and caloric intake. Global *Sk2*, *CG40733* and *dNepYr* RNAi knockdown adult flies were administered 0 uM and 10 uM dRYamide containing liquid food in the CAFE. After 3 days of administration, (A) caloric intake and (B) *dNepYr* mRNA levels were measured. (C) Global *Sply,dNepYr* double knockdowns, were compared side-by-side to global *Sply* and *dNepYr* knockdowns in (D) daily caloric intake and (E) TG levels.

Next, we attempted to pharmacologically rescue elevated caloric intake in *Sk2* RNAi-mediated knockdowns by administering 10 uM dRYamide to liquid food in the CAFE chamber. *Sk2* RNAi knockdowns administered 10 uM dRYamide for 3 days exhibit a nearly 30% reduction in mean daily caloric intake (Figure 7A) and a 50% reduction in dNepYr expression (Figure 7B). *CG40733* knockdowns showed a similar but stronger effect, where caloric intake was reduced ∼38% (Figure 7A) and *dNepYr* expression levels were reduced by ∼80% at the same concentration (Figure 7B). Conversely, *dNepYr* RNAi knockdowns exhibited no reduction in caloric intake (Figure 7A).

Next, we examined whether knockdown of *Sply* could rescue the *dNepYr* RNAi phenotype. To do this, we generated *Sply;dNepYr* RNAi knockdown flies (Figure 7C). *Sply* RNAi mediated knockdowns exhibit ∼32% reduction in caloric intake (Figure 7D). However, *Sply; dNepYr* double knockdowns exhibit a 63% increase in caloric intake (Figure 7D) and elevated TG levels (Figure 7E). This phenotype is nearly identical to that of *dNepYr* RNAi knockdowns. Therefore, these data suggest that *Sply* suppression of caloric intake is non-overlapping and upstream of dNepYr signaling.

Finally, we attempted to pharmacologically rescue caloric intake in *Sk2^KG050894^* flies by administering the stable and potent S1P analog FTY720P [18]. *Sk2^KG050894^* flies fed liquid food containing 10 uM FTY720P for 3 days exhibit a reduction in caloric intake to near control levels (Figure 6E). Interestingly, administration of FTY720P also reduced expression of *dNepYr* in *Sk2^KG050894^* flies to control levels (Figure 6F). Thus, administration of FTY720P to *Sk2^KG050894^* flies mimicked the effect of S1P on suppression of appetite and *dNepYr* expression.

### Double mutant phenotypes are regulated by both Akhpc and dNepYr

We have shown that the exacerbated lean phenotype in *lace^k05305/2^; Sply^05901^* flies was due to the additive effects of reduced fat storage and reduced caloric intake. These flies also showed a 2.5 fold increase in *Akh* mRNA expression, undetectable post-starvation TG levels (Figure S5C), and significantly decreased *dNepYr* mRNA expression levels (Figure S5A). Conversely, obese *ifc^4^; Sk2^KG050894^* double mutants exhibit reduced *Akh* mRNA expression levels (Figure S5B), elevated post-starvation TG levels (Figure S5C) and elevated *dNepYr* mRNA expression levels (Figure S5A). Thus, the additive effects of changes in Akhpc-mediated fat storage mobilization and *dNepYr*-mediated caloric intake underlie the exaggerated phenotypes of the double mutants.

### Sphingolipid metabolite and phenotype correlations

**Figure 8:**
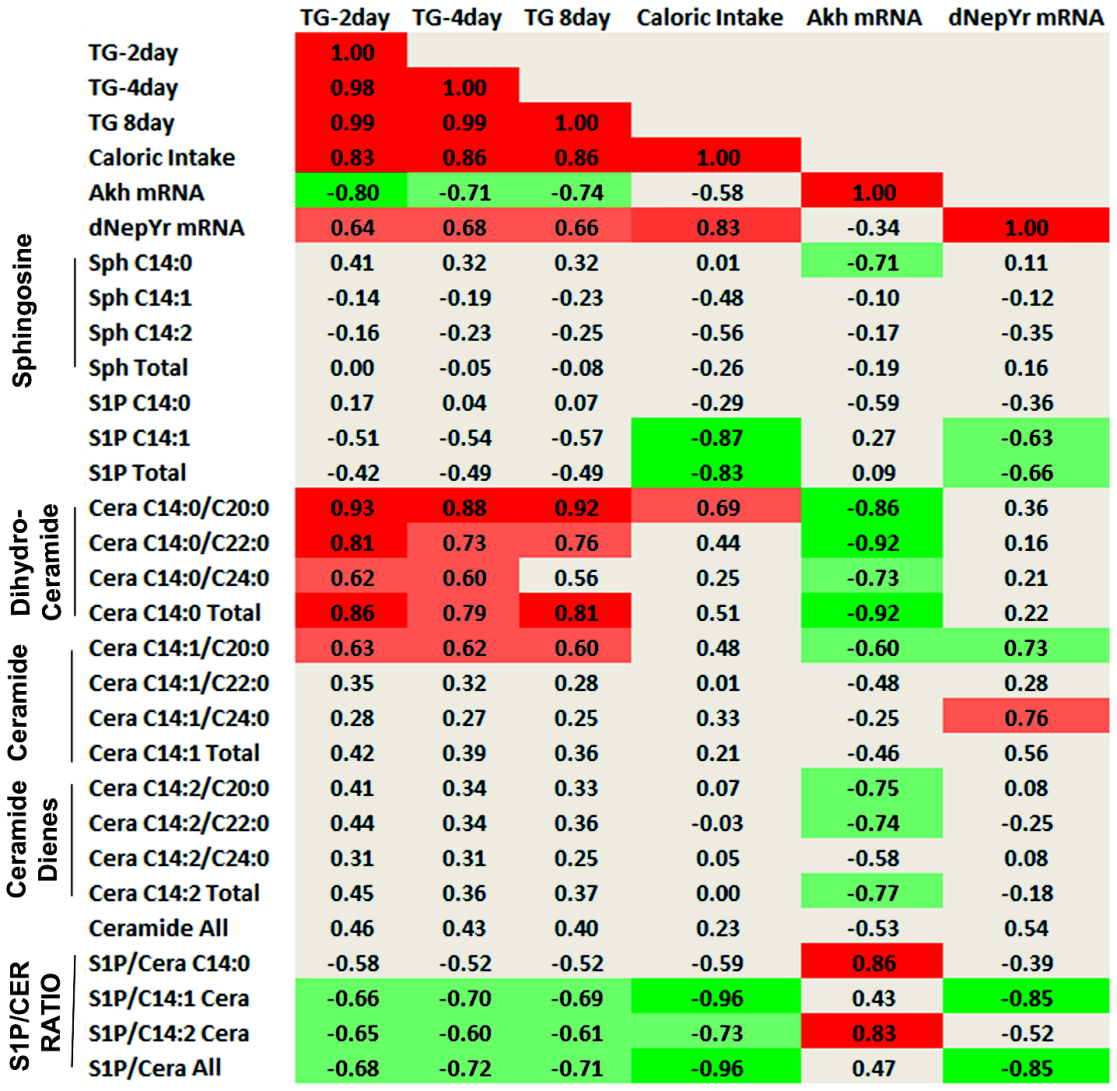
Sphingolipid intermediate and phenotype correlations. Pearson correlation coefficients (multiple r) were calculated between normalized SL metabolites and various metabolic parameters. Dihydroceramide (C_14:0_), ceramide (C_14:1_) and ceramide diene (C_14:2_) subspecies are shown and represent the degree of saturation on the sphingoid backbone. C_20:0_, C_22:0_ and C_24:0_ denote the length and saturation of the second fatty acid chain connected to the sphingoid backbone in these ceramides. The S1P: ceramide ratio is calculated using the sum of total S1P levels over each respective ceramide (C_14:0_,C_14:1_,C_14:2_) as well as the sum of all ceramides (S1P/Ceramide All). Light green denotes negative correlation (p<0.05), bright green denotes strong negative correlation (p<0.01), light red denotes positive correlation (p<0.05), bright red denotes strong positive correlation (p<0.01).

Perturbations of SL genes lead to changes in multiple upstream and downstream SL intermediates. Thus, it is difficult to determine which SL species are most correlated with the observed metabolic phenotypes. In order to identify these associations, we calculated Pearson correlation coefficients (multiple r) between normalized SL intermediate levels and phenotypes (Figure 8).

First, these data show a strong positive correlation between stored TG levels (2, 8, 15 days) and caloric intake (r = 0.83, 0.86, 0.86; p<0.01), as would be expected. Caloric intake also exhibited a strong positive correlation with *dNepYr* mRNA expression levels (r = 0.83; p<0.01). Hence, *dNepYr* mRNA and TG levels were also positively correlated, albeit to a lesser extent (r = 0.64, 0.68, 0.66; p<0.05). Conversely, *Akh* mRNA expression showed a negative correlation with TG levels (r = −0.80, −0.71, −0.74; p<0.05).

The SL's that exhibited the strongest positive correlation with *dNepYr mRNA* expression were C_14:1/C20:0_ and C_14:1/C24:0_ ceramides (r = 0.73, 0.76; p<0.05). Conversely, a strong negative correlation was observed with C_14:1_ S1P (r = −0.63; p<0.05). The ratio of total S1P: C_14:1_ ceramide displayed the strongest negative correlation to *dNepYr* mRNA expression (r = −0.85; p<0.01). Importantly, the S1P: C_14:1_ ceramide ratio also showed a prominent negative correlation with caloric intake (r = −0.96; p<0.01) and to a lesser extent, TG levels (r = −0.68, −0.72, −0.71; p<0.05). Thus, these data establish a relationship between the S1P: ceramide ratio, *dNepYr* mRNA expression, caloric intake and TG stores, which are primary drivers of caloric intake-dependent phenotypes in *Sply*, *Sk2* and their respective double mutants. No significant correlation was observed between sphingosines, dihydroceramides, or ceramide dienes with *dNepYr mRNA* expression levels (Figure 8).

The SL's that exhibited the strongest negative correlation with *Akh* mRNA expression were dihydroceramides (r = −0.92; p<0.01). A negative correlation was also observed with ceramide dienes (r = −0.77; p<0.05). Additionally, dihydrosphingosine (r = −0.71; p<0.05) and to a lesser extent C_14:1/C20:0_ ceramides (r = −0.60; p<0.05) were also negatively correlated. Interestingly, C_14:0_ S1P showed a modest correlation (r = −0.59; p<0.05) with *Akh* mRNA expression. Furthermore, the S1P: C_14:2_ ceramide ratio exhibited a stronger correlation to Akh mRNA expression then C_14:2_ ceramides alone (r = 0.83; p<0.01). Importantly, dihydroceramide species display a significant positive correlation with TG levels (r = 0.86, 0.79, 0.81; p<0.01). These data establish a strong correlation between specific ceramide subspecies, *Akh* mRNA levels and TG stores, which are primary drivers of caloric intake–independent phenotypes in *lace*, *ifc* and their respective double mutants. Additionally, these results suggest that S1Ps could exhibit a modest opposing role in these processes (Figure 8).

## Discussion

### Sphingolipid metabolites regulate lipid storage and feeding behavior

**Figure 9:**
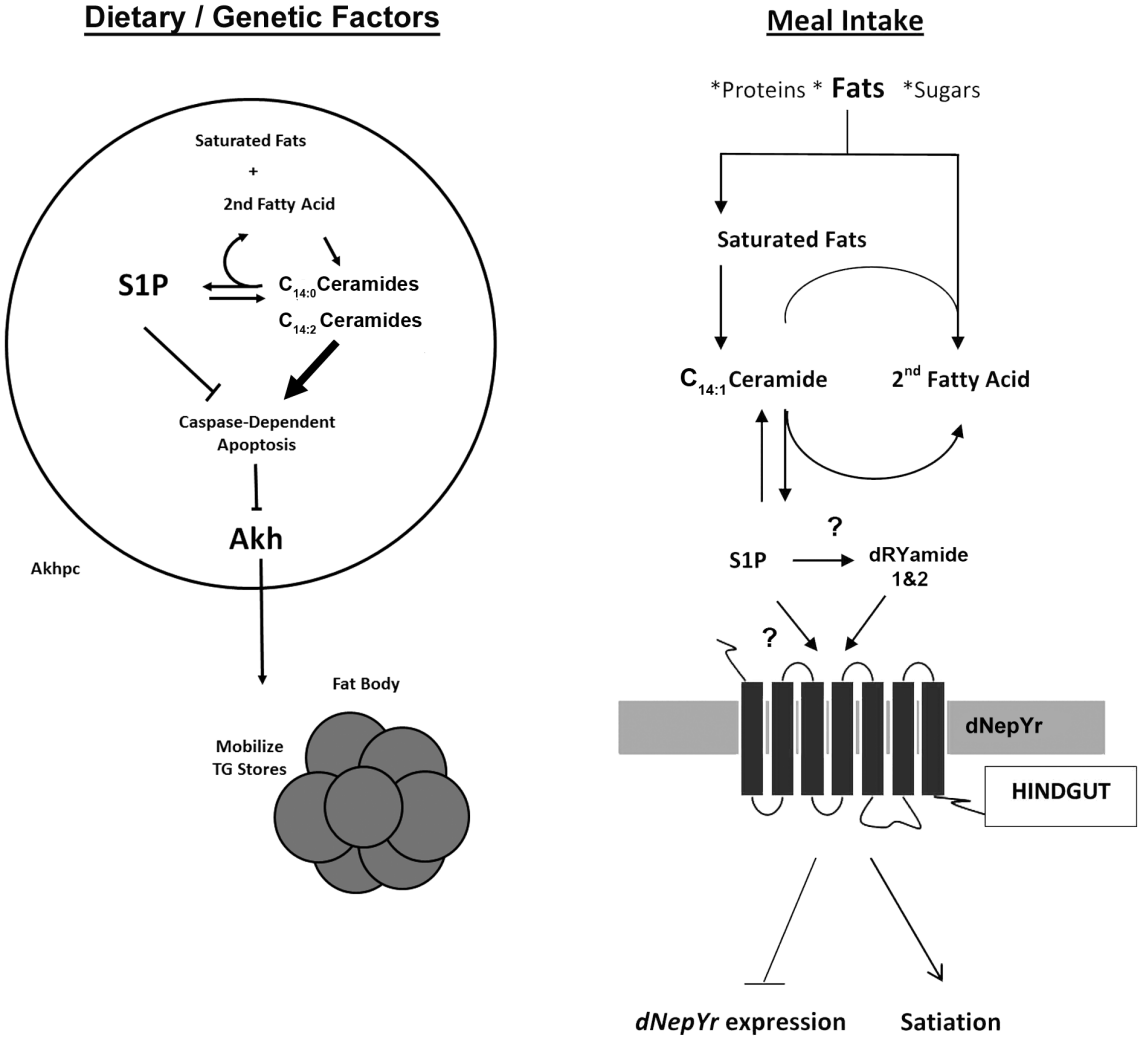
Sphingolipid regulation of caloric intake and fat mobilization. Both saturated and unsaturated fats act as precursor “input” molecules in the production of “output” sphingolipid intermediates, specifically ceramide and S1P, which act to transduce a physiological response. (Left) Sphingolipid metabolism regulates Akh cell viability and function (Right). After a meal, S1P accumulates downstream of elevated ceramide. S1P, either directly or indirectly (through dRYamide), induces dNepY receptor signaling in the hindgut, inducing appetite suppression, reduced caloric intake and downregulation of *dNepYr* mRNA expression (negative feedback).

Using a systems biology approach, we were able to correlate specific changes in SL profiles with changes in metabolic phenotypes and metabolic gene expression. Our results show that the SL intermediates dihydroceramide and ceramide diene are involved in the regulation of fat storage via Akhpc-mediated lipid mobilization. Moreover, the ratio of S1P: ceramide is involved in the regulation of appetite and caloric intake via dNepYr-mediated satiety in flies (Figure 9).

### Comparison with genetic and pharmacological sphingolipid models

#### Serine palmitoyl-CoA transferase

*Lace* mutants exhibit caloric intake-independent leanness in which ceramide depletion is accompanied by enhanced function of Akhpcs associated with a pro-survival gene program, leading to reduced fat storage. Caloric intake-independent weight loss has also been observed in *SPTLC1* mutant mice, which have reduced SPT activity [19]. Furthermore, pharmacological inhibition of SPT activity using myriocin lowered ceramide levels, reduced body weight and fat stores in genetically obese *ob/ob* mice [7]. Our results are also consistent with a previous study in flies, which showed that blocking *de novo* synthesis of ceramide by knocking down *schlank*, a ceramide synthase encoding gene (Figure 1A) downstream of *lace* but upstream of *ifc*, leads to larvae with reduced triglyceride levels relative to controls [20]. Thus, the leptogenic effect of lowering ceramide levels is conserved in mammals and flies.

#### Sphingosine Δ-4 desaturase

Mutant *ifc^4^* flies exhibit caloric intake-independent obesity in which accumulation of distinct ceramide subspecies is accompanied by loss of function of Akhpcs associated with a pro-apoptotic gene program leading to increased fat storage. Genetic studies in mice have shown that homozygous null *DES1* mutants display incomplete lethality [21]. Surviving animals are characterized by severe abnormalities and fail to thrive 8–10 weeks after birth [21]. Heterozygous *DES1* mutant mice have no observable health abnormalities and have wild-type mean body mass and glucose tolerance levels. However, they do exhibit increased insulin sensitivity and resistance to dexamethasone-induced insulin resistance [21].

Interestingly, *DES1* heterozygotes displayed only a slight reduction in ceramide and slight gain in dihydroceramide levels, which constituted a significant reduction in the ceramide: dihydroceramide ratio [21]. No ceramide dienes were reported. This is important to note as particular subspecies of SL intermediates have been shown to exhibit dramatically different biological activities *in vitro*, where degree of saturation can have dramatic impacts on ceramide/sphingosine activity [22]. This implies that it is important to focus not just on the genetic manipulation of SL metabolism, but also on the resulting changes in sphingolipid intermediate levels.

#### Sphingosine kinases

*Sk2^KG050894^* mutant flies exhibit caloric intake-dependent obesity in which ceramide accumulation and S1P depletion are associated with a lipogenic/obesogenic gene program. We showed that orexogenic feeding behavior in these flies stem from the loss of dNepYr mediated satiety signaling. In mice, little is known about any role that sphingosine kinases (SphK) may play in obesity. Both *Sphk*1 and *SphK2* knockout mice are viable, with no observable abnormalities compared to wild type mice [23]. This was attributed in part to redundant function of both enzymes, as S1P levels are only slightly decreased in most tissues of *Sphk1^−/−^* mice [23]. However, *Sphk1^−/−^ Sphk2^−/−^* double knockout mice exhibit undetectable levels of S1P, and do not survive past embryonic day 13.5, due largely to neural and vascular developmental defects [23].

*Sk2^KG050894^* mutant flies do not exhibit compensatory function from Sk1, as S1P levels are nearly undetectable in these flies. Despite the dramatic reduction of S1P, these mutants are viable into adulthood. Interestingly, *Sk2^KG050894^* mutant flies don't appear to exhibit metabolic adaptation to elevated ceramide levels as was recently observed in early adult *dcerk^1^* (ceramide kinase 1) mutant flies [24]. This might explain why early onset of the obese phenotype is observed in *Sk2* but not *dcerk1* mutants. However, differential expression of gut lipases was similarly observed, which likely contribute to the observed obesity phenotype in both *Sk2* and late adult *dcerk1* mutants [24].

#### Sphingosine 1-phosphate lyase

Mutant *Sply* flies exhibit caloric intake-dependent leanness in which S1P accumulation in the context of increased ceramide levels is associated with a lipolytic/leptogenic gene program. We showed that anorexic feeding behavior in these flies' stems from the gain of dNepY receptor mediated satiety signaling.

Comparatively, *SPL* knockout mice (sphingosine 1-phosphate lyase) are also lean and exhibit a pronounced lack of adipose tissue. Interestingly, elevated lipid levels, including TG levels, are observed in both plasma and in fatty livers, reminiscent of human lipodistrophies. However, these mice fail to thrive and do not survive beyond weaning. This has been attributed to multiple abnormalities including congenital defects, anemia, pathological lesions, and a presumed reduction in caloric intake.

Lipodistrophies are difficult to distinguish in the context of flies as the fat body cells share functional homology with both adipose tissue and the liver. However, the fly model is advantageous in that *Sply^05901^* mutants are viable. This allows for the study of the roles of SL metabolites in regulating feeding behavior and lipid metabolism outside of the context of other serious abnormalities throughout development and in adults. It is interesting to speculate that feeding behavior defects in *SPL* mutant neonates might be attributed in part to suppression of appetite via S1P accumulation.

### Sphingolipid metabolism regulates Akhp cell viability and function

Our data suggest that sphingolipid metabolism is involved in regulating Akhpc viability and function via the caspase dependent intrinsic apoptotic pathway. Interestingly, these data also suggest that dihydroceramide and ceramide dienes are likely potent inducers of this pathway and may be involved in the regulation of Akhp cell viability and function. In flies, Akh regulates fat mobilization in the fat body [25]. In mammals, fat is mobilized in adipose tissue by catecholamines and in the liver by glucagon. It will be interesting by analogy to the fly to determine whether SL modulation of ceramide dienes, is involved in the regulation of glucagon production by alpha-pancreatic cells and/or catecholamine production by the adrenal gland.

### dNepYr signaling: A novel sphingolipid-dependent feeding regulatory pathway

Our results showed a substantial reduction in the S1P: C_14:1_ ceramide ratio in *Sk2* mutants. These data correlated with reduction in postprandial satiety via the hindgut-specific neuropeptide like receptor *dNepYr*, resulting in caloric intake-dependent obesity. Furthermore, these data show that dietary administration of dRYamide 1 and 2 was sufficient to overcome these effects through induction of dNepYr signaling. In addition, dietary administration of the S1P analog FTY720P was also effective at inducing satiety and downregulation of *dNepYr* mRNA expression.

Conversely, we showed a substantial increase in the S1P: C_14:1_ ceramide ratio in *Sply* mutants. These flies exhibit suppressed appetites, which also appear to be dependent upon dNepYr. *Sply;dNepYr* double knockdowns exhibited increased caloric intake and TG levels comparable to *dNepYr* knockdowns. These data suggest that S1P acts upstream of dNepYr and induces satiety via dNepYr signaling.

Since *de novo* S1P production is dependent upon the availability of both dietary saturated fats and ATP, it is an attractive candidate molecule for regulating satiety via dNepYr, which is expressed almost exclusively in the hindgut of the closed digestive system. In addition, a set of pancreatic TG lipases, which appear to be overlapping *cis*-NATs of dNepYr, exhibit co-expression patterns and thereby may constitute a coordinated system for dietary fat digestion and satiety.

## Materials and Methods

### Fly husbandry

All fly lines were propagated on synthetic yeast based media as described [8]. The following lines (and relevant CG stock numbers) as listed below: Canton S (wild-type flies), *yw*, *Sply^05901^* (BL-11393), *lace^k05305^* (BL 12176), *lace* ^2^ (BL 3156), *Sk2^k6050894^*, *ifc* ^4^ (BL-1549), UAS-*dIAP* (BL-6657), Global Actin 5c-Gal4 (BL 4414). All double mutants were generated using classic genetics in-house (Herr, et al. 2003). UAS-*dNepYr*-RNAi, UAS-*lace*-RNAi, UAS *akh*-RNAi, UAS-*ifc*-RNAi,UAS-Sk2-RNAi,UAS-Sply-RNAi,and UAS-CG40733-RNAi were acquired through Vienna Drosophila RNAi Stock Center Akh-gal4, UAS-GFP driver lines were graciously provided by the lab of Dr. Ronald Kuhnlein.

### Bioinformatics

Nearest human homologues to fly sphingolipid proteins were determined using protein sequences from Flybase [13]. Blastp results for these sequences against the human proteome were performed at NCBI. Nearest human homologues were determined as the nearest hit based on identity and e-values. The accession number for these proteins was provided. The dNepYr sequence was provided by Flybase. Multiple sequence alignment was performed with ClustalW and the 7TM domain structural motif determined by Chou-Fastman plot.

### Sphingolipidomic profiling

Measurement of major sphingolipid intermediates was performed using liquid chromatography tandem mass spectroscopy (LC/MS/MS) on a Thermo Finnegan triple quadrupole machine. Fly lipid extracts were prepared as previously described [26]. C18 standards of sphingosine, S1P and ceramide (not present in *Drosophila*) were acquired through Avanti Polar Lipids and used as internal standards in positive-mode multiple reaction monitoring (MRM). MRM experiments were adapted from those previously described so that only major sphingolipid species previously identified in Drosophila were monitored, identified, and quantified [27].

### Biochemical assay

Drosophila TG levels were determined using Infinity TAG Reagent kits as previously described [28]. Protein concentrations were determined using the Bradford-Lowry assay. TAG levels were calculated in micrograms of TG per milligrams of protein and then presented as a percentage of the control TG levels. TG levels represent a mean value of triplicate measurements of 50 flies, with corresponding standard deviations, performed six times.

### Starvation resistance assay

Fly stocks were cleared and newly emerged flies collected at the end of 6 hours. Each fly line was sorted into 10 food vials with 10 flies each, 5 males and 5 females, until 2 days of age. Flies were then transferred to agar-vials which provided only a supply of water. Survival rate was determined by the regular counting of non-responsive flies. The experiment was performed in triplicate, with each time point representing the mean based on 300 flies per line. These experiments were adapted from previous studies [9].

### Fat cell measurements

Fat cells were extracted from 1 day old (±6 hours) flies, spilled into Ringer's buffer, fixed with 4% paraformaldehyde, stained with Nile red (10 ug/mL) and DAPI (1 ul/mL). Fat cells were then immediately photographed using a Canon 1500 digital camera on a compound light microscope at 400×magnification. The area of each cell was calculated using ImageJ Imaging Software. Each value represented is the mean of 50 random fat cell measurements taken per fly with the corresponding standard deviation. The experiment was performed in triplicate with the data representing a final mean of 150 cells. Lipid droplet size was assessed in 10 representative fat body cells for each line based on mean fat body cell size. The area of the 10 largest lipid droplets within each cell was measured. A second measure of nile red positive area was performed using a preset color threshold across all FBs and lines. This area was quantified as a % area of the total cell area.

### Feeding assays

#### Starvation-induced appetite response

At three days of age, flies were transferred to agar-only starvation vials for 2 hours. These flies were then transferred into synthetic yeast-based food vial stained with 0.10% Bromophenol Blue. Flies were scored for blue colored abdomens every 30 minutes for 3 hours. Each value represented is the mean of 100 flies taken per fly line with the corresponding standard deviation. The experiment was performed in triplicate with each data point representing a final mean of 300 flies. This experiment is based on variations of the methods described [9].

#### Capillary feeding (Cafe) assay

One week old male flies were raised in a humidity controlled (>70%) incubator at 22°C on a 12 hour day and night cycles. Daily food consumption was measured in microliters per day based on displacement of liquid food levels in a 5 ul microcapillary tube. The experimental setup, procedures and determination of meal frequency were performed as described [10].

#### dRYamide and FTY720P assays

Synthetic yeast based food was allowed to cool to near room temperature and mixed with appropriate concentrations of 1∶1 dRYamide 1: dRYamide 2 peptide before setting. For the CAFE assays, peptide was added to liquid food media described in [10]. These peptides were custom synthesized by Eton Bioscience Inc. according to amidated amino acid sequences which were previously shown to efficiently bind to dNepYr and inhibit feeding behavior responses [17]. FTY720P was also added to liquid media food in the CAFE and acquired from Caymen Chemical.

### Quantitative real-time PCR

Total RNAi was isolated from 25 whole flies with a Qiagen RNeasy Isolation kit as per manufacturer's protocol. Total RNA (1 ug) was checked for quality and purity using gel electrophoresis and UV spectrophotometry. RNA was subsequently reverse-transcribed using an iScript cDNA synthesis Kit from Biorad as per manufacturer's protocol. Primers were designed using Perl Primer software and ordered from ValueGene. Standard PCR was used to test primer sets for single amplicon products. Annealing temperature of the primers was optimized and One-Step qPCR was carried out using a BioRad iCycler IQ. Housing keeping gene RPL-32 was used as a control.

### DNA microarray

RNA was isolated from 25 female flies aged 2 days using a Qiagen RNA isolation Kit. Affymetrix chips were used with oligo targets for 14,131 known *Drosophila* genes. Statistical validation of duplicate experiments results were carried out using Affymetrix software Gene Spring. Heat mapping analysis of metabolic gene subsets was carried out using open source MeV software provided generously to the public by TIGR. The gene subsets selected were based on globally expressed *Drosophila* metabolic genes found to be homologous to mammalian genes by KEGG Pathways using DAVID (david.abcc.ncifcrf.gov/).

### Pearson correlation coefficients

Pearson correlation coefficients (multiple r) were calculated using the excel correlation data function to measure the degree of correlation between normalized SL metabolites and various metabolic parameters, including 2, 8 and 15 day TG levels, caloric intake, *Akh* mRNA expression, and *dNepYr* mRNA expression. Coefficients were determined using relevant data points across all fly lines (wt, *lace*, *ifc*, *Sk2*, *Sply*, *lace*; *Sply*, and *ifc*; *Sk2*) normalized to wt levels. P-values were calculated using a standard t-test.

## Supporting Information

Figure S1 Verification of SL gene expression in P element and RNAi knockdown flies. Data is represented as relative mRNA expression normalized to wildtype flies (canton-s and w^1118^ respectively). Each SL mutant and RNAi KD fly was measured for mRNA expression of their respective target gene. (A–D)P element mutants. (E–H) RNAi-mediated KD. Error bars represent S.E.M. (* = p-value<0.05).
(TIF)

Figure S2 Hallmarks of obesity. Mean triglyceride (TG) levels in (A) SL mutants over deficiency lines and (B) globally driven SL RNAi knockdown flies. (C) Mean body weight (mg/fly). (D)Mean hemolymph TG. (E) Size distribution of lipid droplet size. Red dots denote the largest lipid droplet from each of fat body cell. Red bar denotes their mean. Black dots represents all droplets and black bar denotes their mean.(F)Mean absolute nile red positive area(red) and unstained area (black), with (G) nile red positive area size also represented as a percentage of total FB cell area.
(TIF)

Figure S3 SL mutants and abdominal adiposity. Cryostat sections (30 uM) of flies oriented in a dorsal (left) and sagittal (middle and 60× right) positions. Red = lipid positive oil red o. (A)wt (B) *Sply* (C) *lace* (D) *lace/Sply* (E) *ifc* (F) *Sk2* (G) *ifc;Sk2*.
(TIF)

Figure S4 Characterization of dNepYr and CG40733. (A)The dNepYr gene location, (B) tissue expression profile (www.flybase.org) and (C) expression throughout development. (D) dNepYr mRNA expression in global dNepYr KDs (E) TG levels (ug/mg fly). (F) Caloric Intake in the cafe (ul food/day). (F) Daily caloric intake. CG40733 encodes for RYamide, a known ligand of dNepYr. In global CG40733 KD flies: (G) CG40733 mRNA (H) Caloric intake (ul food/day) and (I) TG levels (ug/mg fly).
(TIF)

Figure S5 Role of dNepYr and akh in SL obesity phenotypes. (A) Relative dNepYr mRNA expression and (B) relative Akh mRNA expression in SL mutants and double mutants. (C) Pre and post starvation TG levels (D)Akh mRNA expression levels in Akh-specific Sply KD flies. (E) TG levels in Akh specific Sply KD flies. (F) Akh mRNA levels in global ifc KD flies.
(TIF)

Figure S6 Adipokinetic hormone producing cells in 3^rd^ instar larvae. Representative 40× images of GFP expressing Akhpc in 3^rd^ instar larvae through the cuticle. First Column is overlayed with DIC. Akh specific gal4 drivers were crossed with UAS RNAi lines. Images include (A) Akh-ga4/+ controls (B) Akh-g4/Akh RNAi (C) Akh-g4/ifc-RNAi (D) Akh-g4/+;UAS-dIAP1 (E) Akh-g4/lace-RNAi (F) % of Larvae that were positive for GFP (G) Area and optical density of GFP expression (H) Akh mRNA expression in 3^rd^ instar larvae.
(TIF)

## References

[1] El Alwani M, Wu BX, Obeid LM, Hannun YA (2006) Bioactive sphingolipids in the modulation of the inflammatory response. Pharmacol Ther 112: 171–183. 16759708 10.1016/j.pharmthera.2006.04.004

[2] Patwardhan GA, Liu YY (2011) Sphingolipids and expression regulation of genes in cancer. Prog Lipid Res 50: 104–114. 20970453 10.1016/j.plipres.2010.10.003 PMC3012148

[3] Jarvis WD, Grant S (1998) The role of ceramide in the cellular response to cytotoxic agents. Curr Opin Oncol 10: 552–559. 9818235 10.1097/00001622-199811000-00013

[4] Hannun YA, Obeid LM (2008) Principles of bioactive lipid signalling: lessons from sphingolipids. Nat Rev Mol Cell Biol 9: 139–150. 18216770 10.1038/nrm2329

[5] Holland WL, Summers SA (2008) Sphingolipids, insulin resistance, and metabolic disease: new insights from in vivo manipulation of sphingolipid metabolism. Endocr Rev 29: 381–402. 18451260 10.1210/er.2007-0025 PMC2528849

[6] Samad F, Hester KD, Yang G, Hannun YA, Bielawski J (2006) Altered adipose and plasma sphingolipid metabolism in obesity: a potential mechanism for cardiovascular and metabolic risk. Diabetes 55: 2579–2587. 16936207 10.2337/db06-0330

[7] Yang G, Badeanlou L, Bielawski J, Roberts AJ, Hannun YA, et al (2009) Central role of ceramide biosynthesis in body weight regulation, energy metabolism, and the metabolic syndrome. Am J Physiol Endocrinol Metab 297: E211–224. 19435851 10.1152/ajpendo.91014.2008 PMC2711669

[8] Herr DR, Fyrst H, Phan V, Heinecke K, Georges R, et al (2003) Sply regulation of sphingolipid signaling molecules is essential for Drosophila development. Development 130: 2443–2453. 12702658 10.1242/dev.00456

[9] Lee KS, You KH, Choo JK, Han YM, Yu K (2004) Drosophila short neuropeptide F regulates food intake and body size. J Biol Chem 279: 50781–50789. 15385546 10.1074/jbc.M407842200

[10] Ja WW, Carvalho GB, Mak EM, de la Rosa NN, Fang AY, et al (2007) Prandiology of Drosophila and the CAFE assay. Proc Natl Acad Sci U S A 104: 8253–8256. 17494737 10.1073/pnas.0702726104 PMC1899109

[11] Fyrst H, Zhang X, Herr DR, Byun HS, Bittman R, et al (2008) Identification and characterization by electrospray mass spectrometry of endogenous Drosophila sphingadienes. J Lipid Res 49: 597–606. 18156591 10.1194/jlr.M700414-JLR200

[12] Gronke S, Muller G, Hirsch J, Fellert S, Andreou A, et al (2007) Dual lipolytic control of body fat storage and mobilization in Drosophila. PLoS Biol 5: e137. 17488184 10.1371/journal.pbio.0050137 PMC1865564

[13] McQuilton P, St Pierre SE, Thurmond J, FlyBase C (2012) FlyBase 101–the basics of navigating FlyBase. Nucleic Acids Res 40: D706–714. 22127867 10.1093/nar/gkr1030 PMC3245098

[14] Chintapalli VR, Wang J, Dow JA (2007) Using FlyAtlas to identify better Drosophila melanogaster models of human disease. Nat Genet 39: 715–720. 17534367 10.1038/ng2049

[15] Parker E, Van Heek M, Stamford A (2002) Neuropeptide Y receptors as targets for anti-obesity drug development: perspective and current status. Eur J Pharmacol 440: 173–187. 12007534 10.1016/s0014-2999(02)01427-9

[16] Graveley BR, Brooks AN, Carlson JW, Duff MO, Landolin JM, et al (2011) The developmental transcriptome of Drosophila melanogaster. Nature 471: 473–479. 21179090 10.1038/nature09715 PMC3075879

[17] Ida T, Takahashi T, Tominaga H, Sato T, Kume K, et al (2011) Identification of the novel bioactive peptides dRYamide-1 and dRYamide-2, ligands for a neuropeptide Y-like receptor in Drosophila. Biochem Biophys Res Commun 410: 872–877. 21704020 10.1016/j.bbrc.2011.06.081

[18] Brinkmann V, Davis MD, Heise CE, Albert R, Cottens S, et al (2002) The immune modulator FTY720 targets sphingosine 1-phosphate receptors. J Biol Chem 277: 21453–21457. 11967257 10.1074/jbc.C200176200

[19] McCampbell A, Truong D, Broom DC, Allchorne A, Gable K, et al (2005) Mutant SPTLC1 dominantly inhibits serine palmitoyltransferase activity in vivo and confers an age-dependent neuropathy. Hum Mol Genet 14: 3507–3521. 16210380 10.1093/hmg/ddi380

[20] Bauer R, Voelzmann A, Breiden B, Schepers U, Farwanah H, et al (2009) Schlank, a member of the ceramide synthase family controls growth and body fat in Drosophila. EMBO J 28: 3706–3716. 19834458 10.1038/emboj.2009.305 PMC2790492

[21] Holland WL, Brozinick JT, Wang LP, Hawkins ED, Sargent KM, et al (2007) Inhibition of ceramide synthesis ameliorates glucocorticoid-, saturated-fat-, and obesity-induced insulin resistance. Cell Metab 5: 167–179. 17339025 10.1016/j.cmet.2007.01.002

[22] Fabrias G, Munoz-Olaya J, Cingolani F, Signorelli P, Casas J, et al (2012) Dihydroceramide desaturase and dihydrosphingolipids: debutant players in the sphingolipid arena. Prog Lipid Res 51: 82–94. 22200621 10.1016/j.plipres.2011.12.002

[23] Mizugishi K, Yamashita T, Olivera A, Miller GF, Spiegel S, et al (2005) Essential role for sphingosine kinases in neural and vascular development. Mol Cell Biol 25: 11113–11121. 16314531 10.1128/MCB.25.24.11113-11121.2005 PMC1316977

[24] Nirala NK, Rahman M, Walls SM, Singh A, Zhu LJ, et al (2013) Survival response to increased ceramide involves metabolic adaptation through novel regulators of glycolysis and lipolysis. PLoS Genet 9: e1003556. 23818862 10.1371/journal.pgen.1003556 PMC3688504

[25] Kuhnlein RP (2010) Energy homeostasis regulation in Drosophila: a lipocentric perspective. Results Probl Cell Differ 52: 159–173. 20865379 10.1007/978-3-642-14426-4_13

[26] Futerman AH, Riezman H (2005) The ins and outs of sphingolipid synthesis. Trends Cell Biol 15: 312–318. 15953549 10.1016/j.tcb.2005.04.006

[27] Merrill AH Jr, Sullards MC, Allegood JC, Kelly S, Wang E (2005) Sphingolipidomics: high-throughput, structure-specific, and quantitative analysis of sphingolipids by liquid chromatography tandem mass spectrometry. Methods 36: 207–224. 15894491 10.1016/j.ymeth.2005.01.009

[28] Gronke S, Mildner A, Fellert S, Tennagels N, Petry S, et al (2005) Brummer lipase is an evolutionary conserved fat storage regulator in Drosophila. Cell Metab 1: 323–330. 16054079 10.1016/j.cmet.2005.04.003

