# Supplementary figures and images for: Identification of Sphingolipid Metabolites That Induce Obesity via Misregulation of Appetite, Caloric Intake and Fat Storage in Drosophila
Source: PLoS Genet. 2013 Dec 5;9(12):e1003970. doi: 10.1371/journal.pgen.1003970 (PMC3854795; doi:10.1371/journal.pgen.1003970)

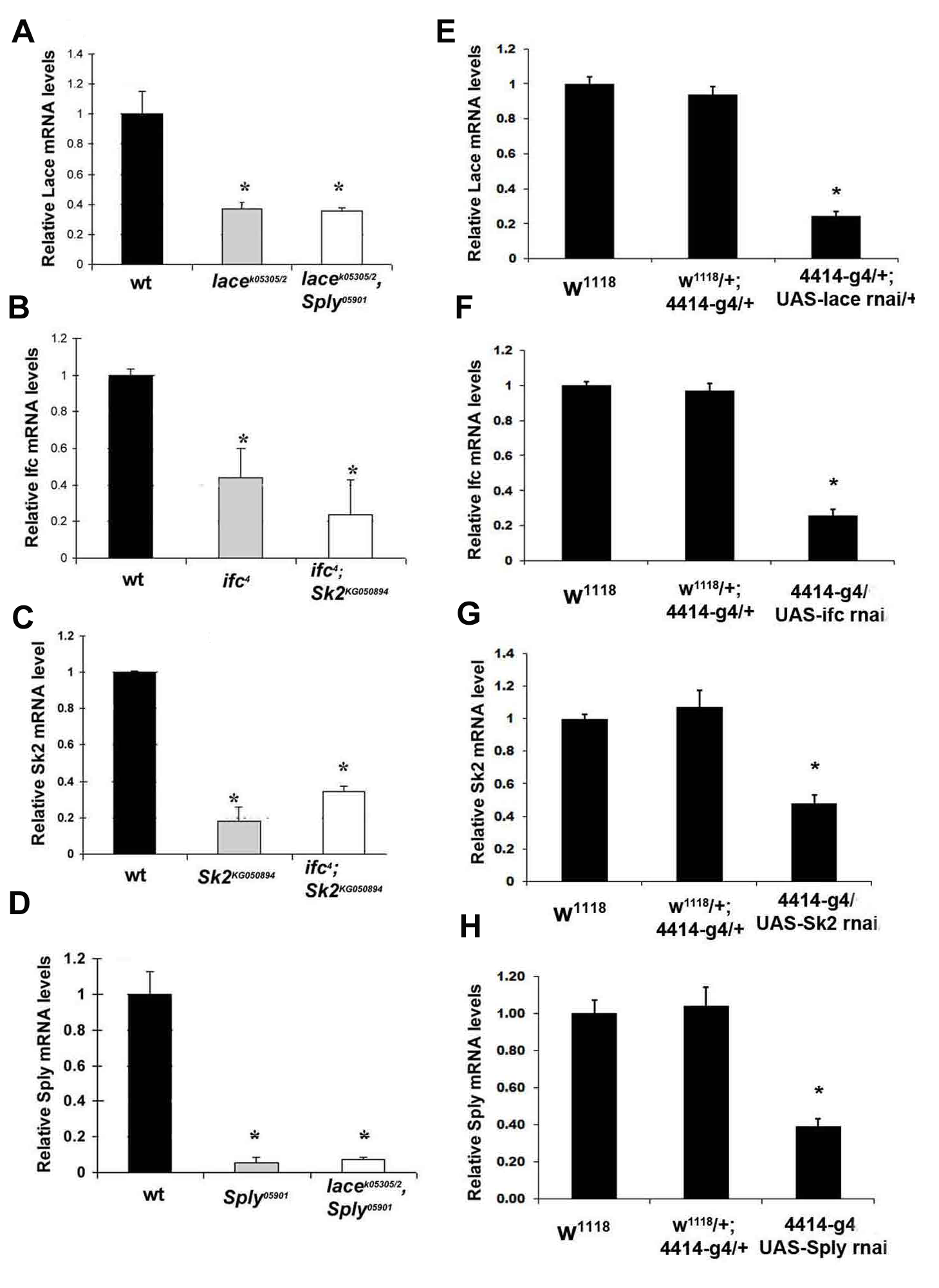

Supplement: Figure S1 — Verification of SL gene expression in P element and RNAi knockdown flies. Data is represented as relative mRNA expression normalized to wildtype flies (canton-s and w1118 respectively). Each SL mutant and RNAi KD fly was measured for mRNA expression of their respective target gene. (A–D)P element mutants. (E–H) RNAi-mediated KD. Error bars represent S.E.M. (* = p-value<0.05). (TIF) [file pgen.1003970.s001.tif]

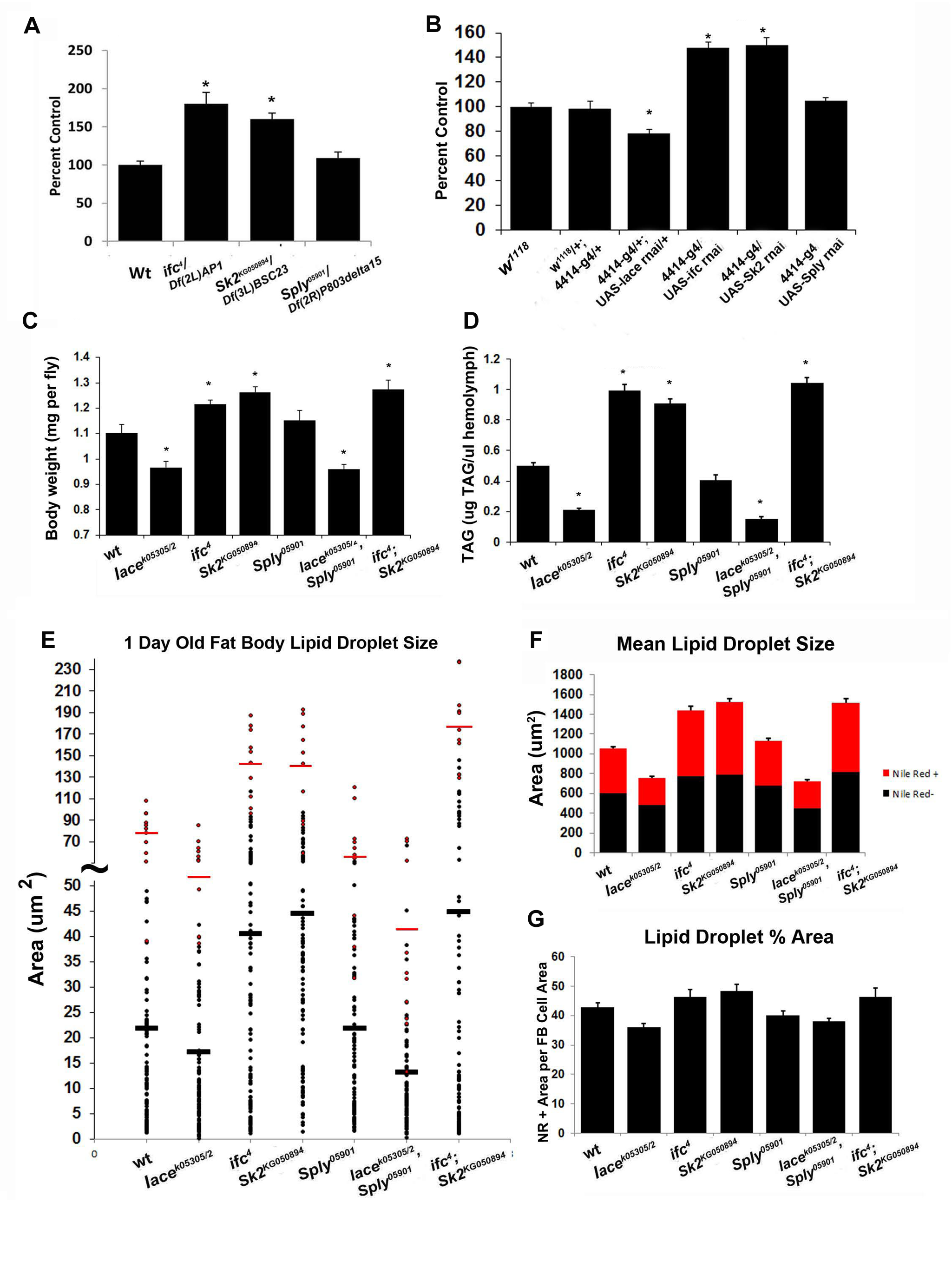

Supplement: Figure S2 — Hallmarks of obesity. Mean triglyceride (TG) levels in (A) SL mutants over deficiency lines and (B) globally driven SL RNAi knockdown flies. (C) Mean body weight (mg/fly). (D)Mean hemolymph TG. (E) Size distribution of lipid droplet size. Red dots denote the largest lipid droplet from each of fat body cell. Red bar denotes their mean. Black dots represents all droplets and black bar denotes their mean.(F)Mean absolute nile red positive area(red) and unstained area (black), with (G) nile red positive area size also represented as a percentage of total FB cell area. (TIF) [file pgen.1003970.s002.tif]

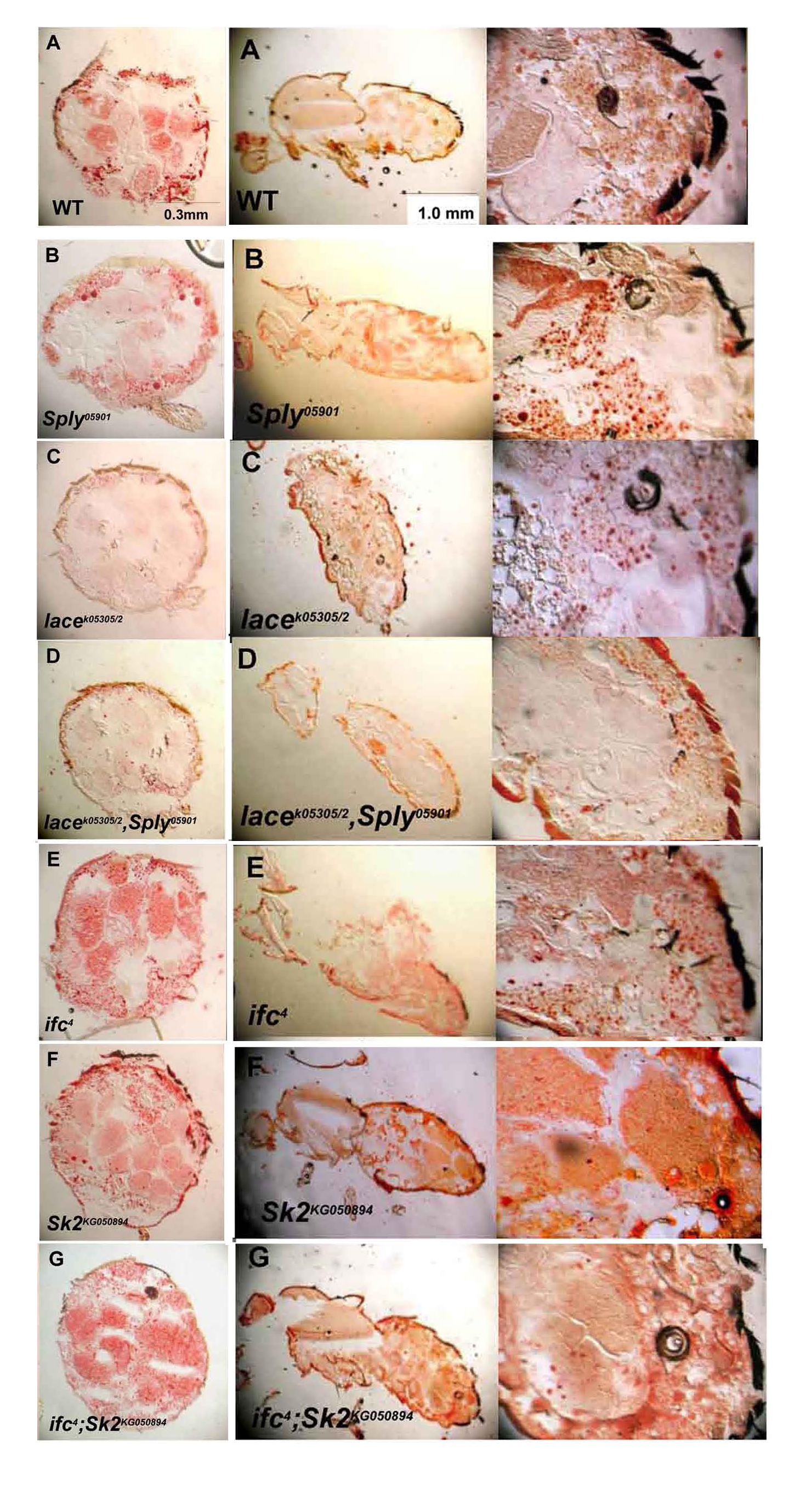

Supplement: Figure S3 — SL mutants and abdominal adiposity. Cryostat sections (30 uM) of flies oriented in a dorsal (left) and sagittal (middle and 60× right) positions. Red = lipid positive oil red o. (A)wt (B) Sply (C) lace (D) lace/Sply (E) ifc (F) Sk2 (G) ifc;Sk2. (TIF) [file pgen.1003970.s003.tif]

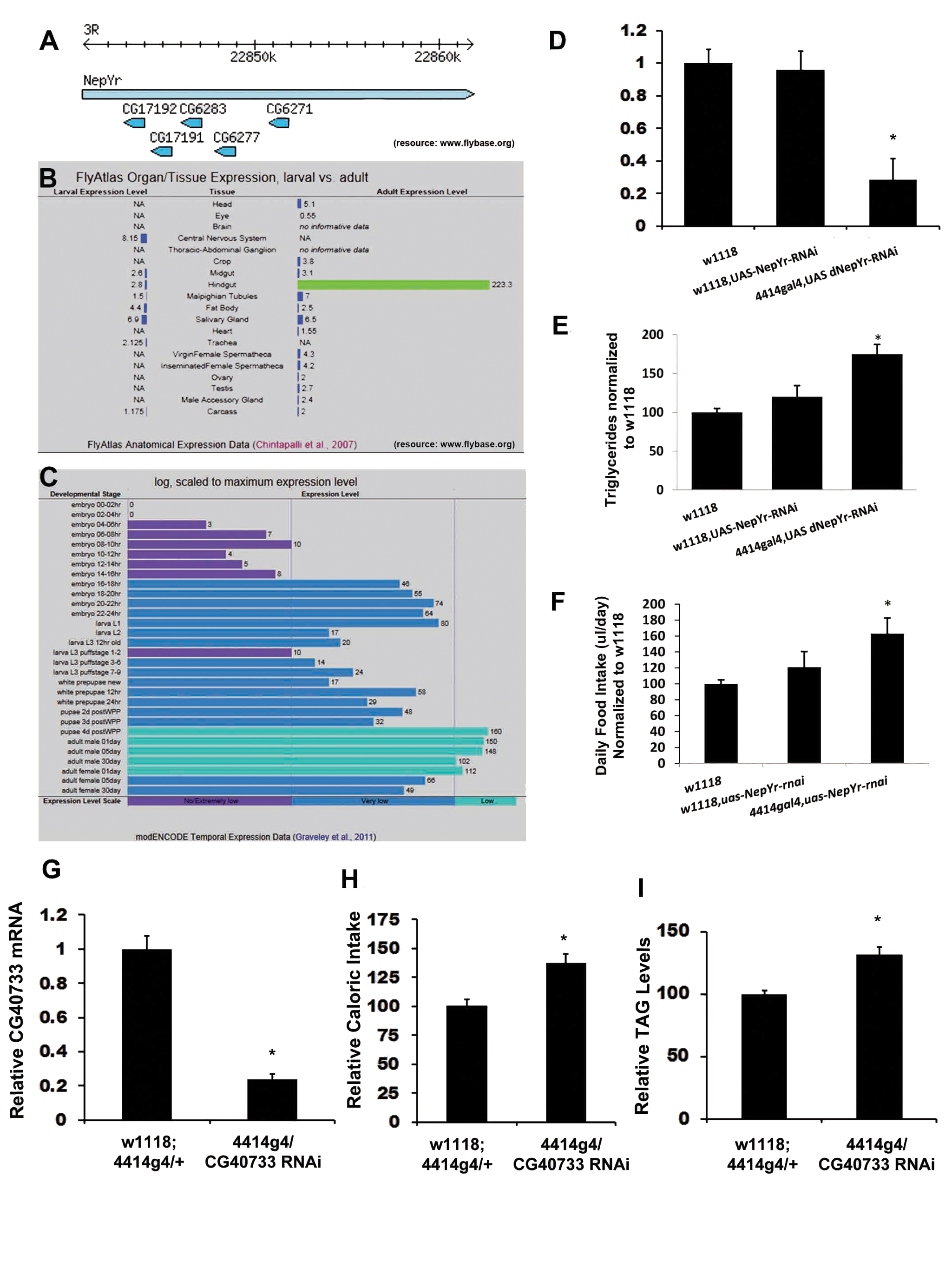

Supplement: Figure S4 — Characterization of dNepYr and CG40733. (A)The dNepYr gene location, (B) tissue expression profile (www.flybase.org) and (C) expression throughout development. (D) dNepYr mRNA expression in global dNepYr KDs (E) TG levels (ug/mg fly). (F) Caloric Intake in the cafe (ul food/day). (F) Daily caloric intake. CG40733 encodes for RYamide, a known ligand of dNepYr. In global CG40733 KD flies: (G) CG40733 mRNA (H) Caloric intake (ul food/day) and (I) TG levels (ug/mg fly). (TIF) [file pgen.1003970.s004.tif]

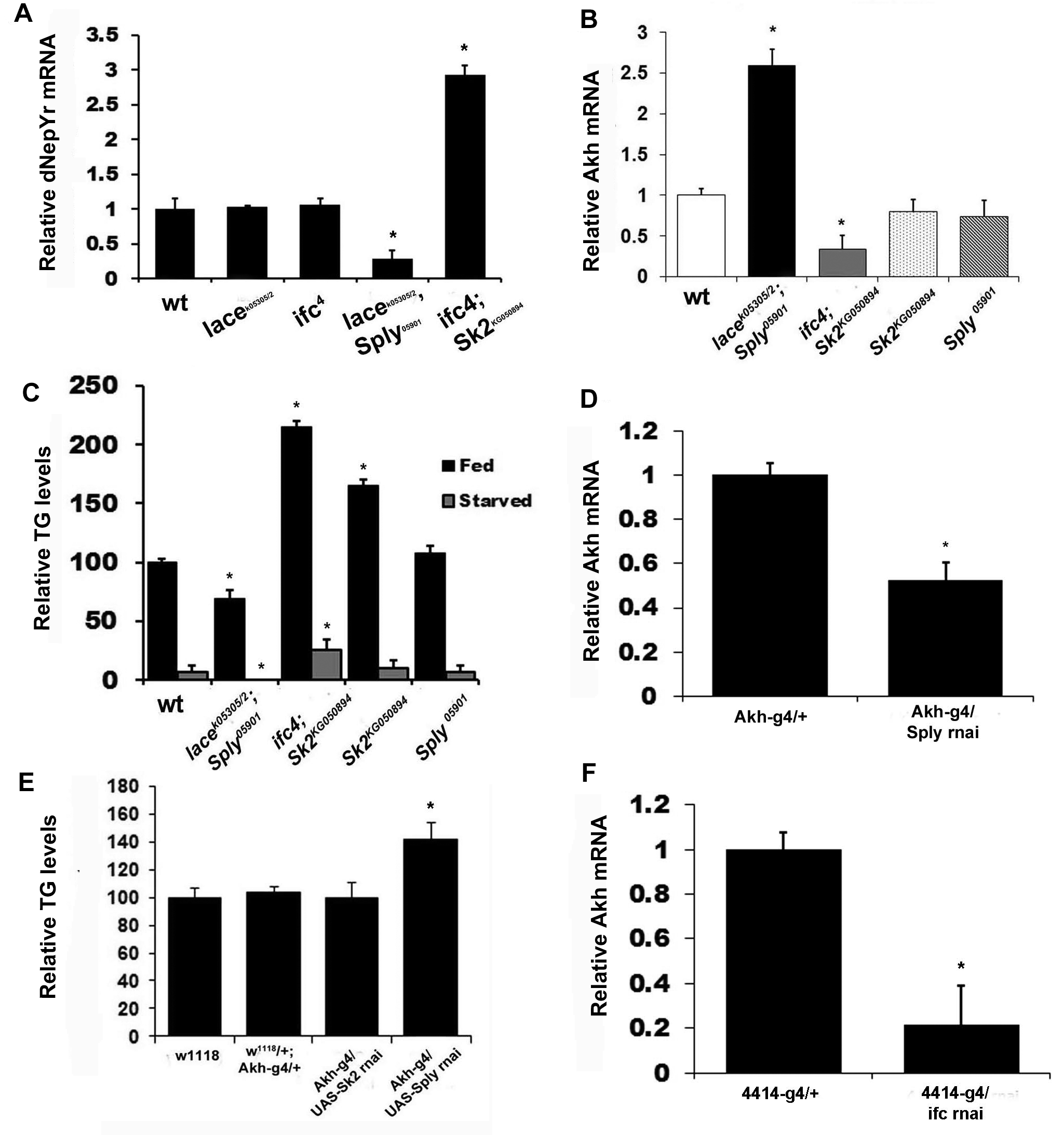

Supplement: Figure S5 — Role of dNepYr and akh in SL obesity phenotypes. (A) Relative dNepYr mRNA expression and (B) relative Akh mRNA expression in SL mutants and double mutants. (C) Pre and post starvation TG levels (D)Akh mRNA expression levels in Akh-specific Sply KD flies. (E) TG levels in Akh specific Sply KD flies. (F) Akh mRNA levels in global ifc KD flies. (TIF) [file pgen.1003970.s005.tif]

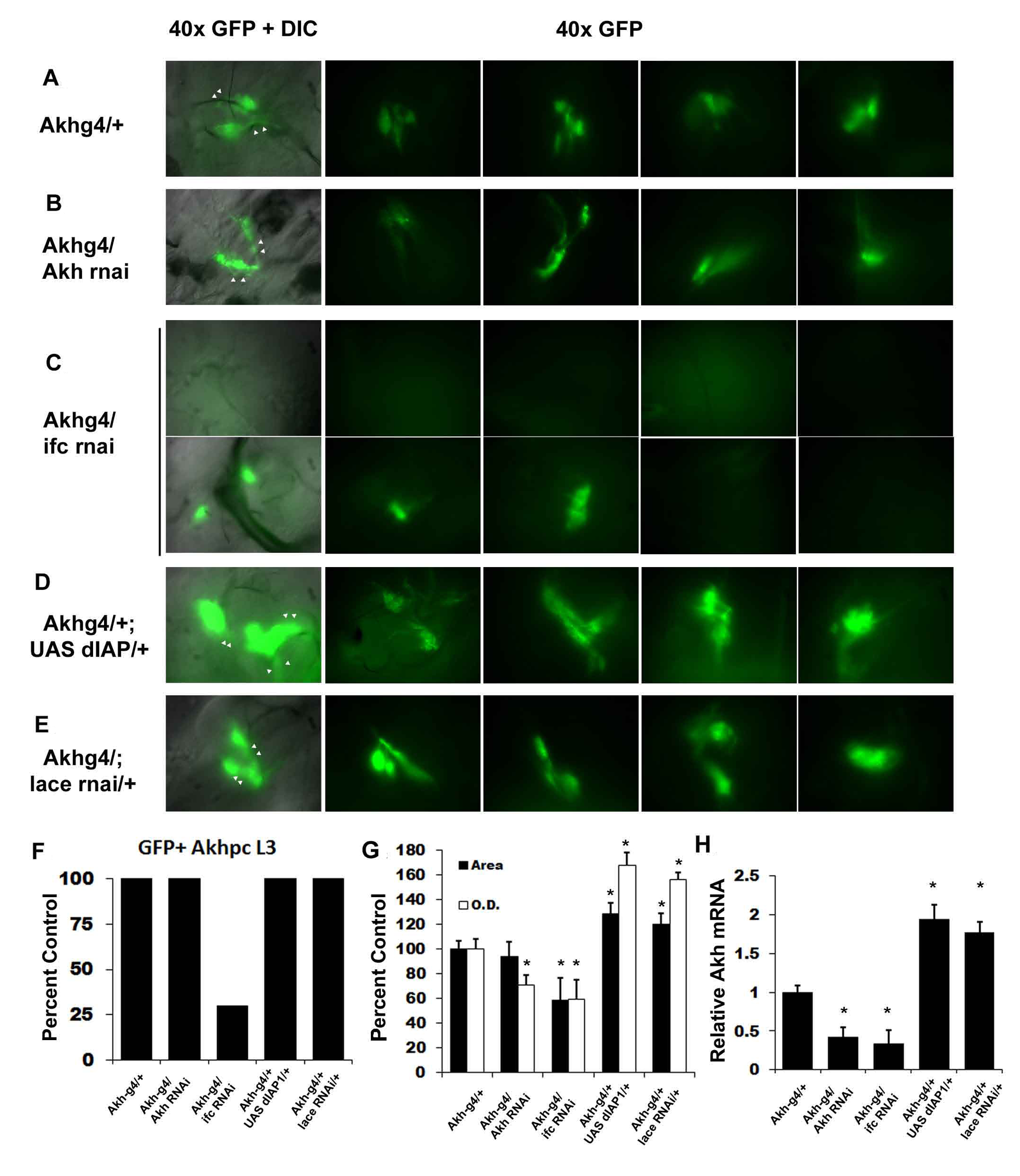

Supplement: Figure S6 — Adipokinetic hormone producing cells in 3rd instar larvae. Representative 40× images of GFP expressing Akhpc in 3rd instar larvae through the cuticle. First Column is overlayed with DIC. Akh specific gal4 drivers were crossed with UAS RNAi lines. Images include (A) Akh-ga4/+ controls (B) Akh-g4/Akh RNAi (C) Akh-g4/ifc-RNAi (D) Akh-g4/+;UAS-dIAP1 (E) Akh-g4/lace-RNAi (F) % of Larvae that were positive for GFP (G) Area and optical density of GFP expression (H) Akh mRNA expression in 3rd instar larvae. (TIF) [file pgen.1003970.s006.tif]
